# [^68^Ga]Ga-NODAGA-TriGalactan, a low molecular weight tracer for the non-invasive imaging of the functional liver reserve

**DOI:** 10.1186/s41181-024-00271-1

**Published:** 2024-05-15

**Authors:** Maximilian A. Zierke, Christine Rangger, Kimia Samadikhah, Marlene Panzer, Stefanie Dichtl, Nikolas Hörmann, Doris Wilflingseder, Andreas M. Schmid, Roland Haubner

**Affiliations:** 1 Department of Nuclear Medicine, Medical University Innsbruck, Anichstr. 35, Innsbruck, 6020 Austria; 2 Werner Siemens Imaging Center, Department of Preclinical Imaging and Radiopharmacy, Eberhard Karls University Tübingen, Röntgenweg 13, 73076 Tübingen, Germany; 3 Department of Internal Medicine I, Medical University Innsbruck, Anichstr. 35, Innsbruck, 6020 Austria; 4 Institute of Hygiene and Medical Microbiology, Medical University Innsbruck, Schöpfstr. 41, Innsbruck, 6020 Austria; 5 Department of Pharmaceutical Chemistry, Institute of Pharmacy, University of Innsbruck, Innrain, 80-82, Innsbruck, 6020 Austria

**Keywords:** Functional liver imaging, Asialoglycoprotein receptor, Positron emission tomography, [^68^Ga]Ga-NODAGA-TriGalactan

## Abstract

**Background:**

Determination of the functional liver mass is important in a variety of clinical settings including liver surgery and transplantation. [^99m^Tc]Tc-diethylenetriamine-pentaacetic acid galactosyl human serum albumin (^99m^Tc-GSA) is a radiotracer targeting the asialoglycoprotein receptor (ASGR) and is routinely used in Japan for this purpose. Here we describe the development and evaluation of [^68^Ga]Ga-NODAGA-TriGalactan a low molecular weight PET-tracer targeting this structure.

**Results:**

For synthesis TRIS as branching unit and NODAGA as chelator for labelling with [^68^Ga]Ga are included. Three galactose moieties are conjugated *via* a click chemistry approach resulting in the desired labelling precursor.^68^Ga-labelling could be accomplished in high radiochemical yield and purity. [^68^Ga]Ga-NODAGA-TriGalactan is very hydrophilic and revealed high plasma stability and low plasma protein binding. Fluorescence imaging showed binding on ASGR-positive organoids and the IC_50_-value was in the nanomolar range. Most importantly, both biodistribution as well as animal imaging studies using normal mice demonstrated high liver uptake with rapid elimination from all other organs leading to even higher liver-to-background ratios as found for ^99m^Tc-GSA.

**Conclusion:**

[^68^Ga]Ga-NODAGA-TriGalactan shows high in vitro stability and selectively binds to the ASGR allowing imaging of the functional liver mass with high contrast. Thus, our first generation compound resulted already in an alternative to ^99m^Tc-GSA for imaging the functional liver reserve and might allow the broader use of this imaging technique.

**Supplementary Information:**

The online version contains supplementary material available at 10.1186/s41181-024-00271-1.

## Background

The asialoglycoprotein receptor (ASGR) is a C-type lectin mainly expressed on the basolateral side of hepatocytes, where up to 500,000 receptors per cell can be found (D’Souza & Devarajan 2015). In contrast, in the remainder of the body expression is low making it a promising target for drug delivery into hepatocytes. Nevertheless, its main physiological function is to maintain serum glycoprotein homeostasis by clearing of proteins bearing desialylated d-galactose (Gal) or *N*-acetylgalactoseamine (GalNAc) as terminal carbohydrates. Therefore, these glycoproteins are internalized via receptor-mediated endocytosis, followed by degradation of the ligands within the lysosome (Stockert 1995).

The ASGR consists of two homologous subunits, namely H1 and H2, which are responsible for Gal/GalNAc recognition (Lee et al. 1983). Carbohydrate binding was elucidated by X-ray structure analysis of the carbohydrate recognition domain (CRD) of the H1 subunit (Meier et al. 2000). It was shown, that binding affinity increases in the order of mono-, di-, and tri-antennary ligands by addressing 2–3 binding sites simultaneously. It was found that these binding sites are 25–30 Å apart (Khorev et al. 2008). Binding assays with synthetic carbohydrates in combination with NMR and molecular modelling studies showed that the optimal arrangement of the terminal sugar moieties of corresponding ligands is a triangle with an edge length of 15, 20, and 25 Å, respectively (Lee et al. 1983). Based on these structural findings trivalent small molecule carriers have been developed showing selective uptake into hepatocytes (Khorev et al. 2008).

Non-invasive methods for functional liver assessment are of great interest for patient management in a diversity of clinical settings including liver surgery and transplantation (de Graaf et al. 2010; Kaibori et al. 2011; Hoekstra et al. 2013) as well as diagnosis (Virgolini et al. 1990) and treatment monitoring (Virgolini et al. 1993) of cancer. Additionally, it has been revealed that the evaluation of remnant liver function can help to discriminate different stages of alcoholic liver cirrhosis (Virgolini et al. 1991) and could be used to differentiate areas of steatosis, fibrosis and cholestasis (Bennink et al. 2012).

Initially, ^99m^Tc-GSA (Kokudo et al. 2003) has been developed to image ASGR expression using single photon emission tomography (SPECT). It has been proven that ^99m^Tc-GSA and dynamic SPECT allow an estimation of regional hepatic function based on the determination of the ASGR density (Kudo et al. 1993). In order to combine the superior performance of positron emission tomography (PET) regarding image resolution and quantification tools with the targeting properties of ^99m^Tc-GSA, we developed a ^68^Ga-labelled analogue (Haubner et al. 2013). Finally, our studies resulted in [^68^Ga]Ga-NOTA-GSA (NOTA = 1,4,7-triazacyclononane-1,4,7-triacetic acid) which showed comparable pharmacokinetics and even higher metabolic stability as the reference compound (Haubner et al. 2017). Despite the good imaging performance of human GSA-based radiotracers the translation of these molecules into clinical practice is limited due to regulations of biological products isolated from human material. Therefore, particular research interest has arisen in the development of galactose based organic small molecule radiotracers. Recently, [^68^Ga]Ga-NOTA-HexaLac a hexavalent lactoside based tracer was introduced for targeting the ASGR (Yu et al. 2018). The preliminary imaging data indicate that, in contrast to GSA-based radiotracers, also low molecular weight compounds can be used for imaging of this receptor. Here we describe the synthesis and evaluation of [^68^Ga]Ga-NODAGA-TriGalactan, a novel PET radiopharmaceutical for imaging the functional liver mass based on a trivalent small molecule carrier.

## Materials and methods

A detailed synthetic procedure for preparation of NODAGA-TriGalactan including analytical data as well as a complete list of all reagents can be found in the supplementary information. Protocols for determination of *logD*, protein binding and stability studies have been described elsewhere (Hörmann et al. 2020).

### ^68^Ga-labelling of NODAGA-TriGalactan

A previously published procedure for labelling of NODAGA-conjugates with gallium-68 was applied (Knetsch et al. 2011). Therefore, 5 nmol (5 µL, 1 mM) of precursor were mixed with 100 µL of a 1 M Na-acetate buffer (pH 5), followed by addition of 550 µL ^68^Ga-eluate (40–50 MBq). This mixture was incubated for 10 min at 56 °C and the radiochemical purity was determined using radio-HPLC and radio-TLC.

### In vitro binding studies

Inhibitory constant (IC_50_) values were determined in triplicates using freshly isolated mouse hepatocytes (Seglen 1976). A detailed protocol can be found in the supplementary information. In brief, cells were incubated in presence of [^125^I]I-asialoorosomucoid (5 nM) with various ligand concentrations ranging from 10^− 5^-10^− 11^ M for 1 hour on ice. Cells were washed and cell bound activity was released by addition of 200 µL 1 M NaOH for 10 min. The activity content in the lysates was quantified in a γ-counter (2480 Wizard^2^ 3”, PerkinElmer, Waltham, Massachussetts, USA) and the values were plotted in excel. Fitting of the sigmoidal binding curve and the calculation of the IC_50_ value was done with excel’s solver plugin.

### Mouse hepatic organoid culture

Mouse hepatic organoids (Stemcell Technologies, Vancouver, Canada) were cultivated in HepatiCult® organoid growth medium according to the distributor’s recommendations. A detailed procedure can be found in the supplementary information.

### Organoid uptake assay and fluorescence imaging

In a 96-well v-bottom plate (Thermo Scientific, Waltham, Massachusetts, USA) mouse hepatic organoids were incubated with 0.3 nM [^68^Ga]Ga-NODAGA-TriGalactan (2.5 kBq per well; A_m_ = 35–45 MBq/nmol) for 1 h at 37 °C in organoid growth medium + 5 mM CaCl_2_. For blocking, the experiment was conducted in presence of 10 mM GalNAc. At the end of the incubation time, cells were put on ice for 1 min and spun down (300 rcf, 5 min, 4 °C). The supernatant was carefully removed and organoids were washed twice with 200 µL of ice-cold PBS. Internalized activity was released by treating the organoids with 200 µL of 1 M NaOH for 10 min. Lysates were transferred into scintillation vials and the activity content was measured in a γ-counter. Organoid uptake was determined as the percentage of total radioactivity added.

For microscopic studies organoids were incubated with 333 nM BP-Fluor-647-TriGalactan for 1 h at 37 °C. Cells were pelleted, washed once with PBS and fixated by addition of 200 µL of fixation buffer (BD Cytofix/Cytoperm™, Fisher Scientific, Hampton, USA). After 1 h at room temperature, cells were washed again with PBS and incubated in 100 µL of perm/wash-buffer (Biozym, Hessisch Oldendorf, Germany) for another 30 min. For counter staining organoids were incubated over night at 4 °C with 30 µL of a mixture of 2 µM Hoechst 33,342 (Thermo Scientific), 165 nM Phalloidin CruzFluor™ 555 (Santa Cruz, Dallas, Texas, USA) and Anti-ASGR1 AF488-coupled antibody (2 µg/mL, Bio-Techne, Dublin, Ireland). The next day, organoids were centrifuged, washed three times with PBS and transferred to a CellCarrier-96 Ultra Microplate (PerkinElmer). The Operetta CLS system (revvity, Hamburg, Germany) was used to image the samples. Analysis was performed using the Harmony software 4.9.

### Biodistribution studies

All animal experiments were conducted in accordance with the Austrian animal experiments law (BGBl. I Nr. 114/2012) and according to the institution’s animal welfare standards as approved by the Austrian Federal Ministry of Education, Science and Research (BMBWF, 2022 − 0.311.708). For biodistribution studies 6 week old female BALB/c mice (*n* = 3, Charles Rivers Laboratories, Sulzfeld, Germany) were injected with 0.1 nmol (100 µL, approx. 1 MBq) of ^68^Ga-labelled compound *via* the lateral tail vein and sacrificed by cervical dislocation 10, 30, or 60 min post injection (p.i.). The mice were dissected, blood and organs were weighed and the activity of the samples measured in a γ-counter. For blocking experiments, mice were co-injected with 27.7 µmol of d-galactose.

### Small animal imaging

All animal experiments were performed according to the German animal welfare act and approved by the local authorities (R5/19). Male C57BL/6J mice were purchased from Janvier Lab (Saint-Berthevin Cedex, France) at 6 weeks. Mice were kept under 1.5% isoflurane in oxygen during the measurements (*n* = 3). Approximately 1 MBq of the radiotracer was injected intravenously per mouse and 1 h dynamic PET was performed with an Inveon microPET system (Inveon D-PET, Siemens, Knoxville, TN, USA). Mice were then transferred to a 7 Tesla BioSpec 70/30 USR (Bruker Biospin MRI GmbH, Ettlingen, Germany) and a 15 min MR anatomical scan using T2-weighted 3-dimensional turbo spin-echo sequence was acquired. An Inveon Acquisition Workplace was used to reconstruct the acquired images using ordered subset expectation maximization 3D (OSEM 3D) and framing the dynamics. The reconstructed image analysis was performed by using an Inveon Research Workplace. After co-registering acquired MR images with PET images, regions of interest (ROIs) were drawn on liver, heart, muscle, and kidney and time activity curves (TACs) were quantified.

### Statistical analysis

Statistical significance of experimental data was calculated in SPSS using student’s *t-*test for unpaired samples.

## Results

### Chemical synthesis

**Scheme 1:**
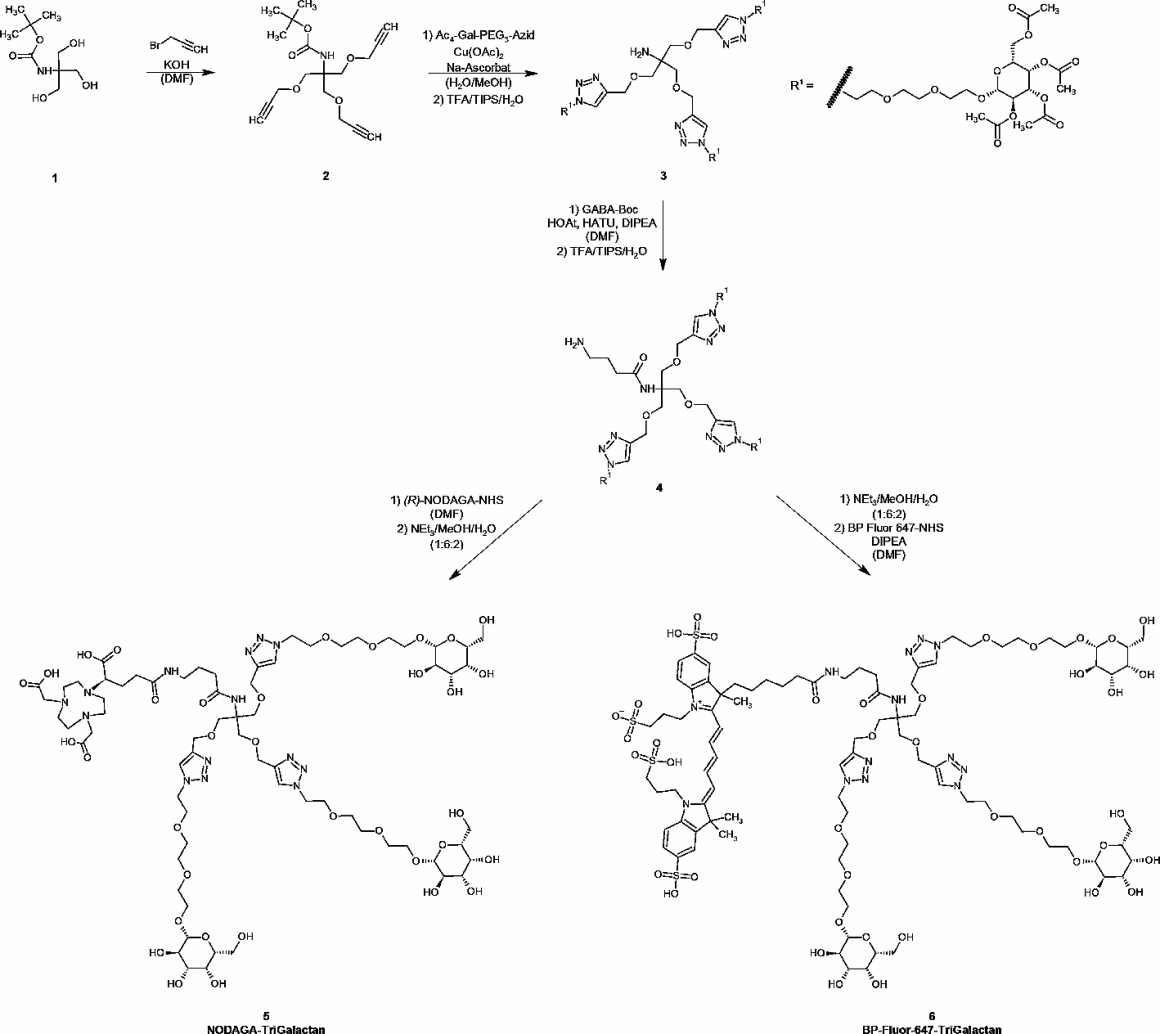
Synthesis of NODAGA-TriGalactan (**5**) & BP-Fluor-647-TriGalactan (**6**)

The labelling precursor NODAGA-TriGalactan was obtained in a seven step synthesis at an overall yield of 3.2% (Scheme 1). For the first reaction step Boc-protected tris(hydroxymethyl)aminomethan (TRIS) (**1**) was reacted with propargyl bromide to give the respective ether conjugate (**2**). Next, Cu-catalyzed click chemistry was used to attach three galactose moieties to the backbone followed by removal of the Boc-protection group using trifluoroacetic acid (TFA) (**3**). 4-Amino butyric acid was introduced as a linker on the free amino function of TRIS (**4**). Coupling of *R*-NODAGA and subsequent removal of all acetyl protection groups yielded NODAGA-TriGalactan (**5**) in a purity of 97.5%. A fluorescent derivative (**6**) was obtained by de-acetylating trimer **4** and reacting it with BP-Fluor-647 NHS-ester.

### Gallium-labelling procedures

**Fig. 1:**
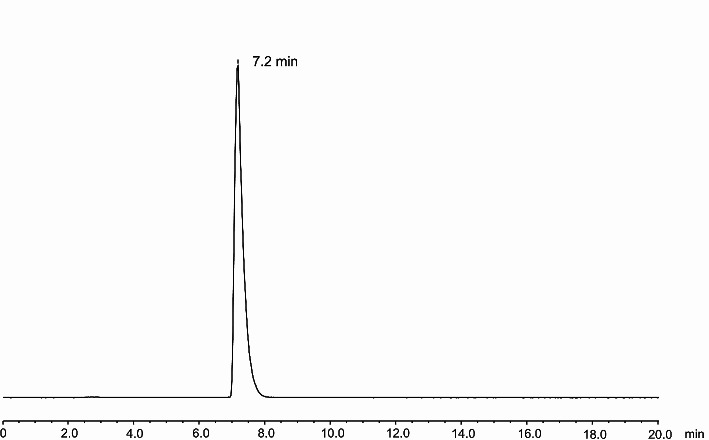
Radio-HPLC of [^68^Ga]Ga-NODAGA-TriGalactan; Column: Reprosil Pur C_18_ AQ 150 × 4.6 mm; Gradient: 5–60% acetonitrile/0.1% TFA in 15 min; t_R_ = 7.2 min

Radiolabelling of NODAGA-TriGalactan with gallium-68 was achieved in high radiochemical yields and purity as determined by radio-HPLC (> 99%) and radio-TLC (> 98%) (Fig. 1). Molar activities were usually between 8 and 10 MBq/nmol. Labelling with naturally occurring gallium (3-fold molar excess based on precursor) happened instantly and led to formation of [^nat^Ga]Ga-NODAGA-TriGalactan in quantitative yields and high purity (> 97%) as determined by HPLC and MS analysis.

### In vitro evaluation

[^68^Ga]Ga-NODAGA-TriGalactan showed high stability in PBS (> 99% intact tracer) and human blood serum (> 93%) over the time course of 2 h (Table 1). Furthermore, low protein binding could be observed, rising from 7.6 ± 3.9% (*n* = 3) after 2 min to 13.6 ± 3.2% (*n* = 3) after 120 min of incubation. Determination of *logD-*values for [^68^Ga]Ga-NODAGA-TriGalactan (− 4.33 ± 0.09, *n* = 6) and the reference ligand ^99m^Tc-GSA (-1.88 ± 0.02, *n* = 8) revealed high hydrophilicity of the trimer. Binding to the ASGR was quantified in an assay with freshly isolated mouse hepatocytes using the ^nat^Ga-complex of NODAGA-TriGalactan. Hepatocytes were seeded in a 24-well plate and competitive binding against [^125^I]I-asialorosomucoid (ASOR) was studied. For comparison, the affinity of GSA for the receptor was also determined. [^nat^Ga]Ga-NODAGA-TriGalactan showed medium high affinity (IC_50_ = 73.2 ± 67.5 nM, *n* = 5) for the receptor. For GSA an IC_50_-value of 19.6 ± 17.1 nM (*n* = 4) was found.

**Table 1:** IC_50_, *logD*, stability in human blood serum (*n* = 2), PBS stability and protein binding (*n* = 3) of ^99m^Tc-GSA and [^68^Ga]Ga-NODAGA-TriGalactan

	^99m^Tc-GSA				[^68^Ga]Ga-NODAGA-TriGalactan			
IC_50_ (nM)	19.6 ± 17.1 (*n* = 4)				73.2 ± 67.5 (*n* = 5)			
*logD*	-1.88 ± 0.02 (*n* = 8)				-4.33 ± 0.09 (*n* = 6)			
	**2 min**	**30 min**	**60 min**	**120 min**	**2 min**	**30 min**	**60 min**	**120 min**
Serum stability (%)	82 ^(Haubner et al. 2013)^	76 ^(Haubner et al. 2013)^	70 ^(Haubner et al. 2013)^	65 ^(Haubner et al. 2013)^	99.8 ± 0	97.6 ± 2.3	98.5 ± 1.2	92.9 ± 4.6
PBS stability (%)	97.6 ^(Haubner et al. 2013)^	97.1 ^(Haubner et al. 2013)^	-	95.6 ^(Haubner et al. 2013)^	99.8	99.8	99.8	99.8
Protein binding (%)	-				7.6 ± 3.9	5.5 ± 0.5	9.2 ± 0.2	13.6 ± 3.2

**Fig. 2:**
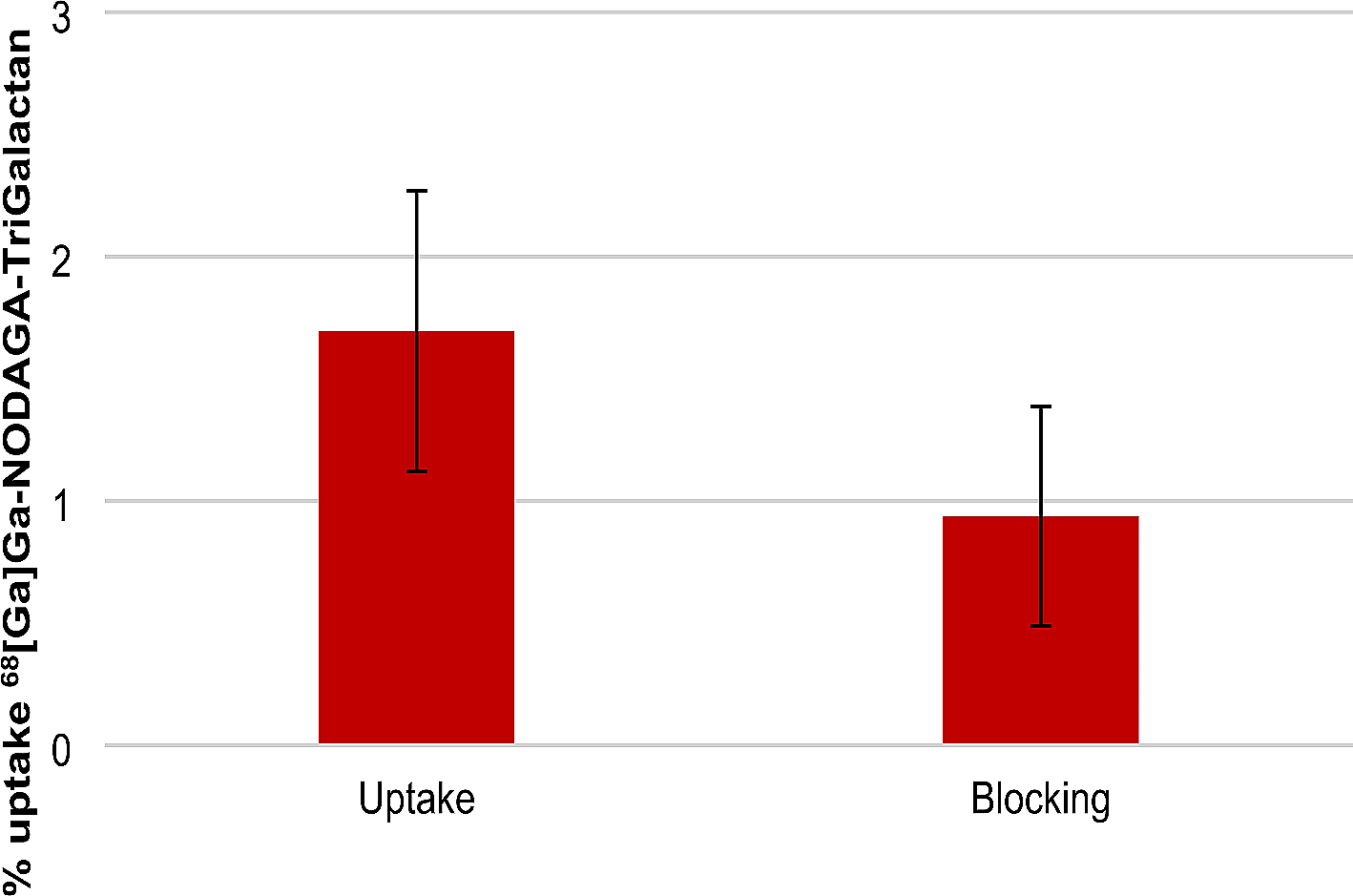
Cellular uptake of ^68^Ga[Ga]-NODAGA-TriGalactan with and without blocking on mouse hepatic organoids after 1 h at 37 °C

In addition, cellular uptake of [^68^Ga]Ga-NODAGA-TriGalactan was studied on mouse hepatic organoids (Fig. 2). Compared to conventional 2D experiments with either HepG2 or HuH-7 cells, organoids have the advantage to represent a complex 3D tissue model in a dish. The tracer showed a decent degree of internalization and blocking the interaction with an excess of *N*-acetylagalctose indicated receptor specific interaction with the ASGR (*p* < 0.05).

**Fig. 3:**
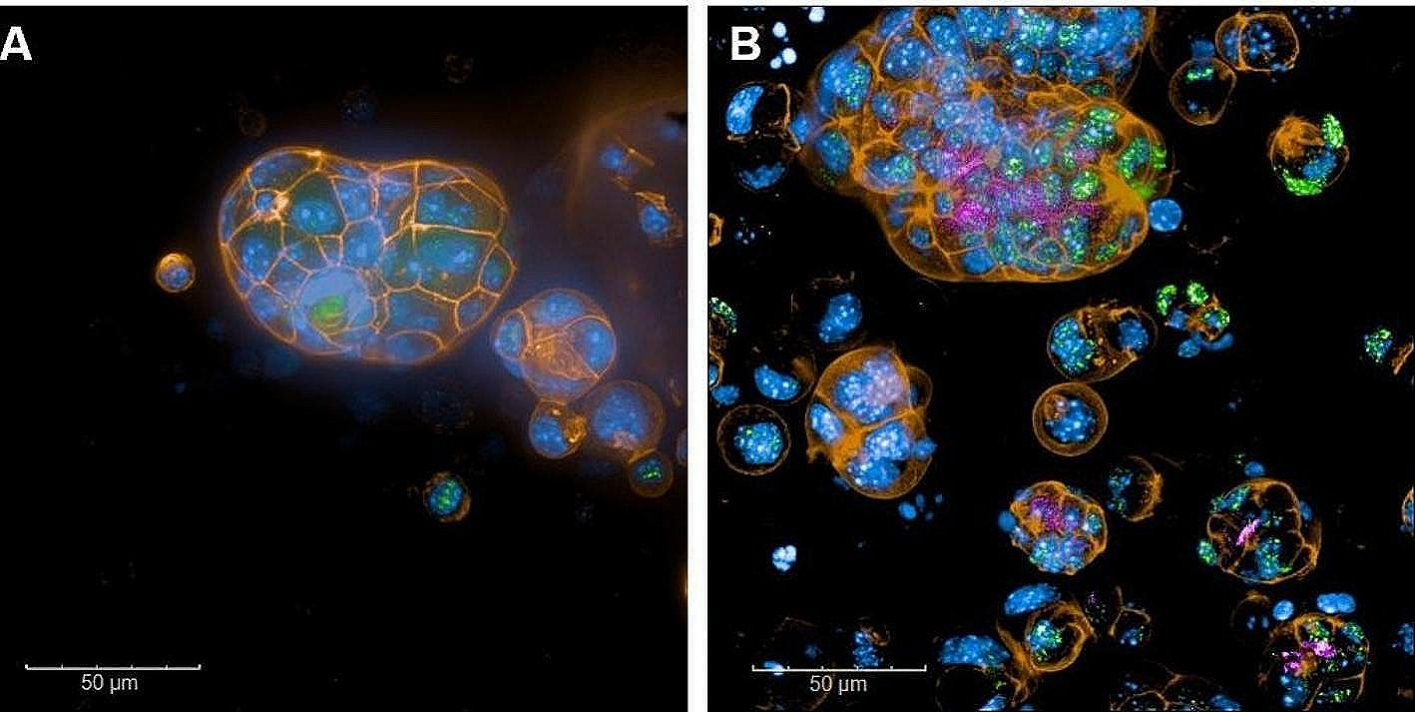
Maximum projection images (25 μm) of mouse hepatic organoids in absence (A) or in presence (B) of BP-Fluor-647-TriGalactan (pink). Nuclei (blue) were stained with Hoechst, cytoskeleton (orange) was stained with Phalloidin, ASGR1 expression (green) was visualized with an anti-ASGR1 antibody

Target expression on mouse hepatic organoids was visualized by fluorescence imaging with an AF-488 labelled anti-ASGR antibody (Fig. 3A). Co-staining of the cells with BP-Fluor-647-TriGalactan, a fluorescent analogue of NODAGA-TriGalactan, confirmed indeed internalization of the trimer into the organoids (Fig. 3B).

### In vivo evaluation

**Fig. 4:**
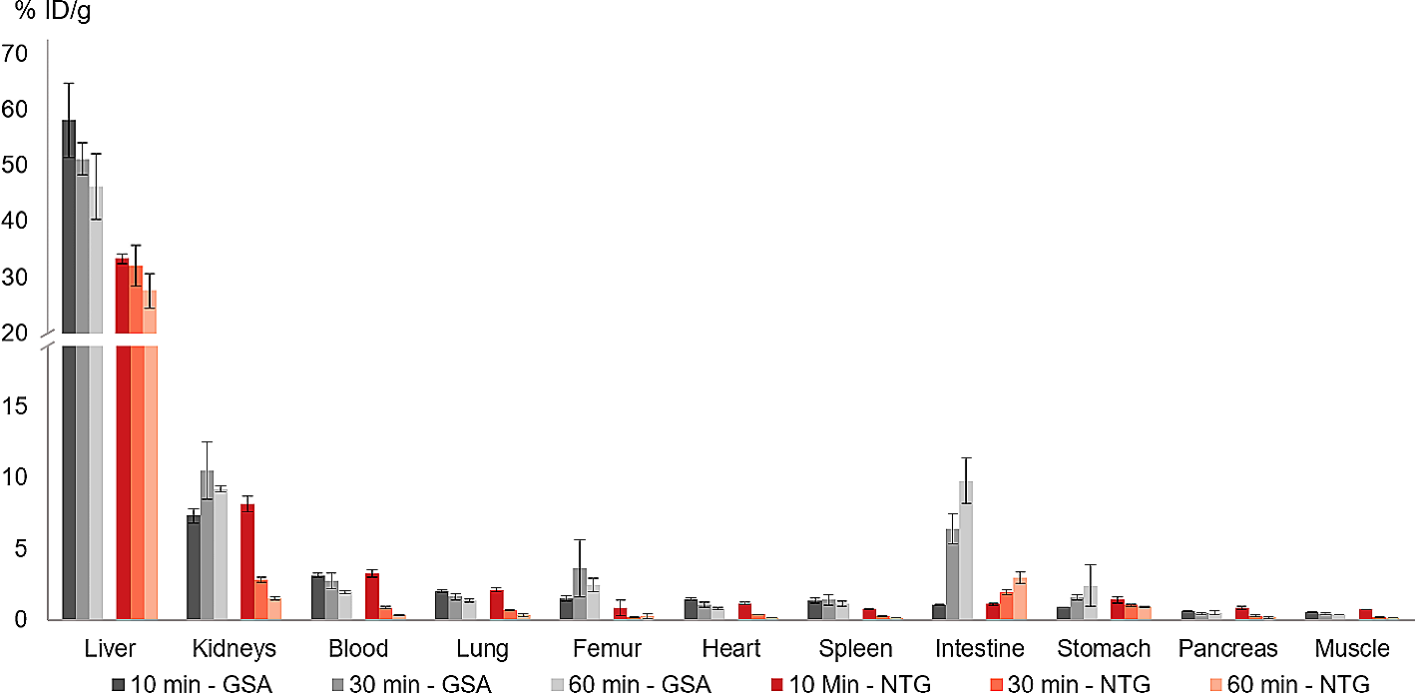
Biodistribution data (% ID/g) of [^68^Ga]Ga-NODAGA-TriGalactan (NTG) (*n* = 3) and ^99m^Tc-GSA (*n* = 3) in healthy BALB/c mice 10, 30 and 60 min p.i. (100 pmol, 1 MBq)

**Fig. 5:**
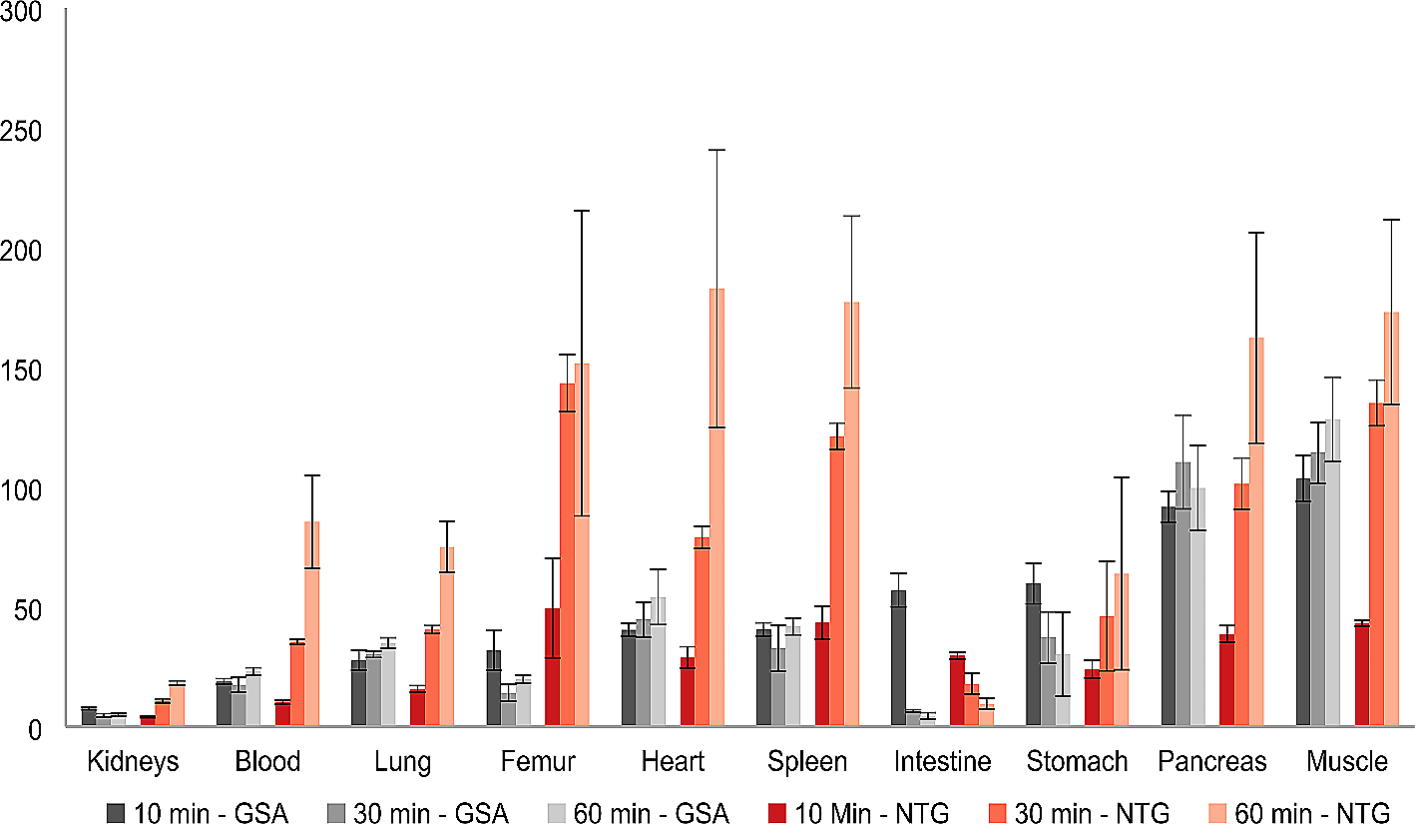
Liver-to-organ ratios for [^68^Ga]Ga-NODAGA-TriGalactan (NTG) and ^99m^Tc-GSA 10, 30 and 60 min p.i. (100 pmol, 1 MBq)

Biodistribution studies of [^68^Ga]Ga-NODAGA-TriGalactan showed high liver uptake reaching its maximum already at 10 min p.i. (33.4 ± 0.9% ID/g) and remaining at a constant high level up to 60 min p.i. (27.7 ± 3.1% ID/g) (Fig. 4, Table S1). Activity elimination from blood was rapid with a blood activity concentration of 0.3 ± 0.04% ID/g 60 min p.i. This results in low non-liver organ uptake. Highest uptake in non-target organs could be found in the kidneys 10 min p.i. (8.2 ± 0.5% ID/g). However, even here the liver-to-organ ratio was increasing from 10 min (4.1 ± 0.4) to 60 min p.i. (18.3 ± 0.9). Most favourable liver-to-organ ratios were reached after 60 min p.i. in almost all organs including blood, spleen, pancreas, heart, lung and muscle demonstrating the described lack of ASGR expression in non-liver tissue and the good pharmacokinetic properties of this new compound (Fig. 5, Table S2). The rapid reduction of activity in kidneys indicated fast renal excretion of the tracer. The only organ with increasing activity concentration over time was the intestine reaching 3.0 ± 0.4% ID/g 60 min p.i.

**Fig. 6:**
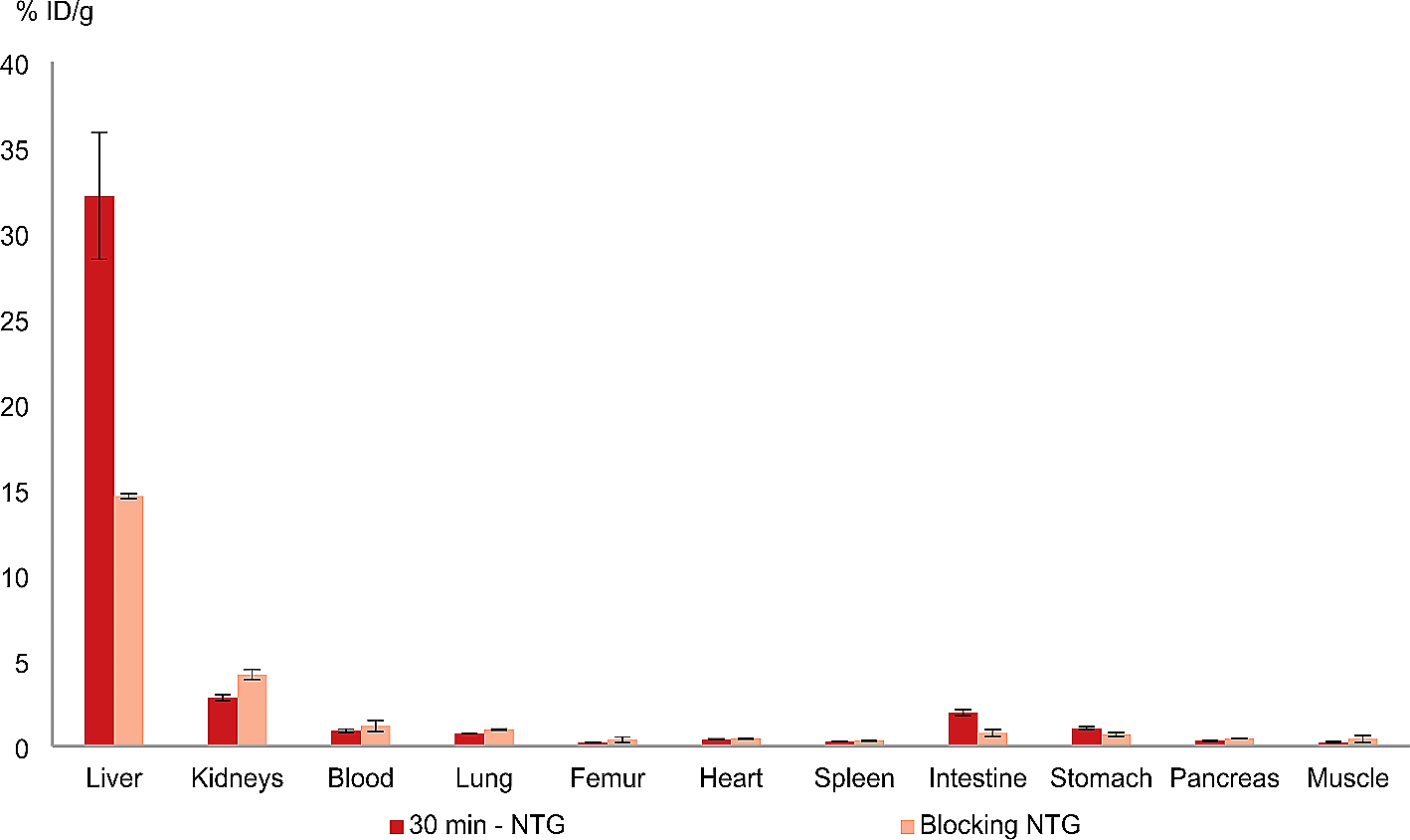
Biodistribution data (% ID/g) of [^68^Ga]Ga-NODAGA-TriGalactan (NTG; *n* = 3) in healthy BALB/c mice 30 min p.i. and at 30 min blocked with 27.7 µmol d-galactose

Blocking experiments were carried out by co-injecting a high excess of d-galactose and sacrificing the mice 30 min p.i. (Fig. 6). A significant reduction in activity accumulation in the liver was found (32.2 ± 3.7% ID/g vs. 14.6 ± 0.15% ID/g) whereas in most non-target organs comparable activity accumulation was observed. Only stomach and intestine revealed an activity reduction where uptake might be effected due to elimination of the high galactose amount and not due to specific interaction with the tracer. Reduced activity concentration in the intestine might also be based on the reduced activity found in liver, which might result in a lower hepatobiliary elimination.

The reference compound ^99m^Tc-GSA showed higher liver uptake 10 min p.i. (58.1 ± 6.7% ID/g) which declined faster over the observation period of 60 min (46.3 ± 5.9% ID/g) (Fig. 4, Table S1). In contrast to [^68^Ga]Ga-NODAGA-TriGalactan, elimination from the blood pool was much slower and activity concentration in kidneys remains almost stable over the observation period of 60 min. Again, highest non-target uptake was found in the kidneys. The increase of activity in the intestine was also higher compared to the new tracer resulting in 9.8 ± 1.6% ID/g vs. 3.0 ± 0.4% ID/g 60 min p.i. In general, activity concentration in observed organs and tissue was higher as found for [^68^Ga]Ga-NODAGA-TriGalactan resulting in inferior liver-to-organ ratios at later time points (Table S2).

### In vivo imaging

**Fig. 7:**
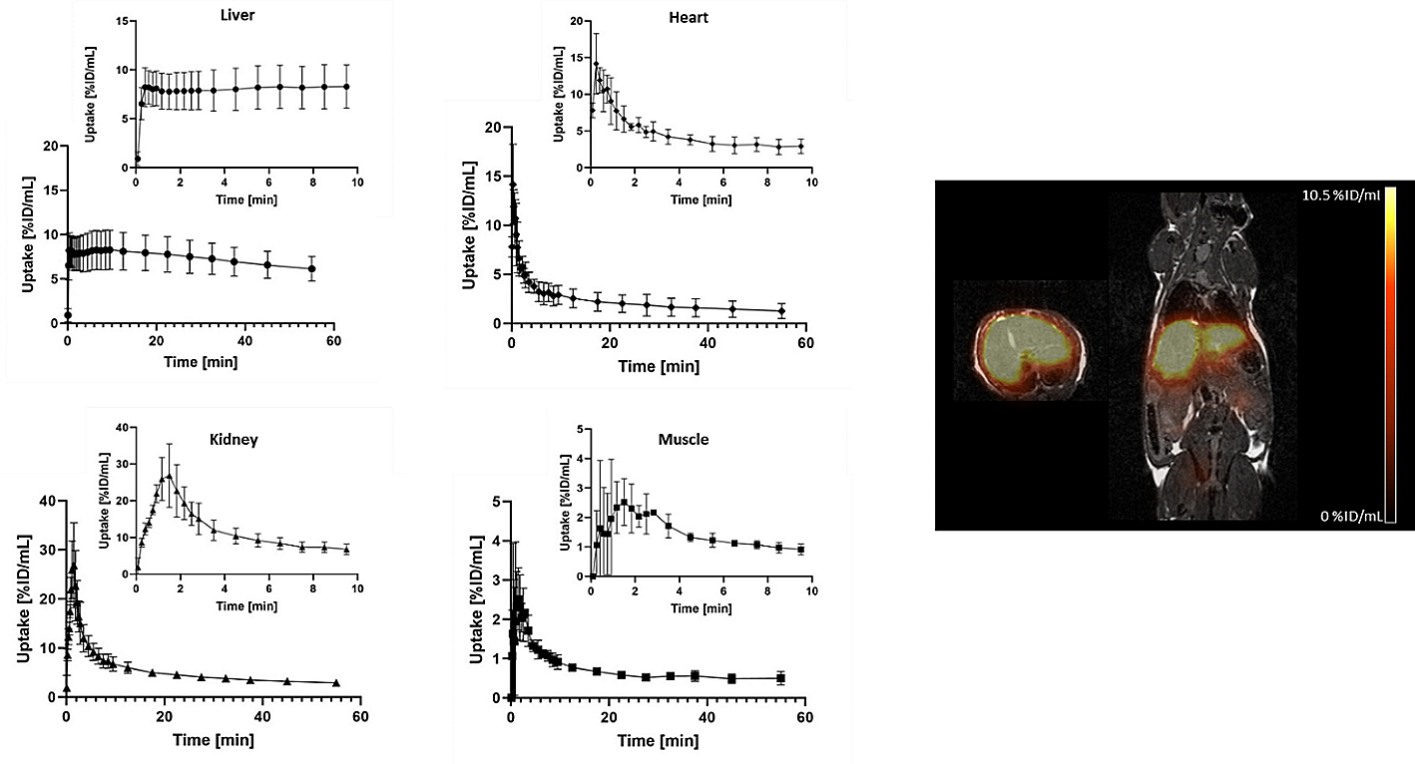
Right: PET/MR fusion of [^68^Ga]Ga-NODAGA-TriGalactan (300 pmol, 1 MBq) in a healthy C57BL/6 mouse, sum of all frames. Left: Corresponding TACs (% ID/mL) of [^68^Ga]Ga-NODAGA-TriGalactan from liver, heart, kidney, and muscle (*n* = 3)

All three mice exhibited analogous uptake patterns of [^68^Ga]Ga-NODAGA-TriGalactan with minimal background activity (Fig. 7). After an initial rise in the first minute, the tracer remained relatively stable in the liver followed by a small washout starting 15 min p.i.. Activity in the blood (measured in the heart) decreased below 5% ID/mL already within the first three minutes. The elimination of the tracer was tracked through the renal system, and the kidneys peaked at ∼ 1.5 min followed by continuous decrease for the rest of the experiment. Background muscle uptake was less than 3% ID/mL.

## Discussion

Since the ASGR has been discovered in the 1960s by Ashwell & Morell (Morell et al. 1968) a variety of research groups have intensively studied carriers and ligand systems for this target structure (Valentijn et al. 1997; Kim et al. 2001; Managit et al. 2005). However, until today research regarding the ASGR is mainly focused on developing probes for targeted delivery of antisense oligonucleotides or drugs to the liver (Li et al. 2008; Prakash et al. 2014). Additionally, galactose–dye conjugates have been reported to allow studies on the underlining receptor mechanism in vitro (Sanhueza et al. 2017). Attempts to find a structure that can specifically target the ASGR for functional imaging using nuclear medicine techniques started with the development of [^99m^Tc]Tc-Neogalactosyl albumin in the mid 1980’s (Vera et al. 1984), whose further optimization led to ^99m^TcGSA (Torizuka et al. 1991). Both tracers feature a serum albumin backbone that has been synthetically galactosylated. Therefore, both ligands are by nature high molecular weight compounds making them hard to be fully characterized. Furthermore, both ligands are designed for labelling with ^99m^Tc, which destines them to be „SPECT-only“ imaging agents. In order to gain access to more synthetically tailored molecules, which can also be applied for PET imaging, the search for small molecule-based liver imaging agents began.

One of the first tracers of this compound class, was 2-deoxy-2-[^18^F]fluoro-d-galactose, a monomeric galactose unit that can be labelled with ^18^F in analogy to [^18^F]FDG (Fukuda et al. 1986). This concept was later reused by Sun et al. who reported a monomeric galactose derivative, that can be labelled with ^18^F *via* click chemistry (Sun et al. 2020). However, both compounds seemed to lack affinity for the ASGR as they only showed little liver uptake in in vivo studies, which limited their further clinical use. In addition, Stokmaier and colleagues focused on monomeric *N*-acetylgalactose derivatives as GalNAc is reported to have 10-times higher affinity for the ASGR than galactose (Stokmaier et al. 2009). Within their study, they replaced the *N*-acetyl group of GalNAc by different organic scaffolds and then evaluated the influence of the structural change on the affinity towards ASGR. They were able to increase the affinity of one GalNAc derivative by a factor of two. However, this approach didn’t result in a suitable high affinity probe for functional liver imaging either.

The general problem with monomeric ligands for the ASGR is the fact, that monomers are lacking the possibility to address all three carbohydrate recognition domains of the receptor simultaneously. This phenomenon, which is termed as the „cluster effect“, is a key factor in designing high affinity probes for the ASGR (Connolly et al. 1981).

**Fig. 8:**
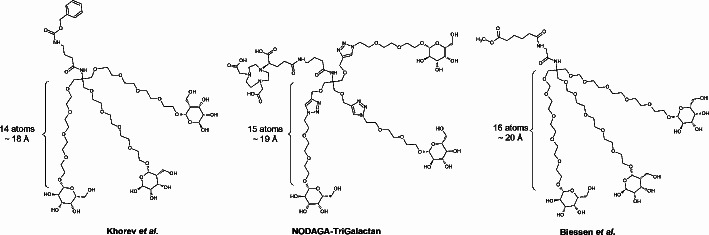
Structures and linker lengths of different galactose trimers compared to NODAGA-TriGalactan

The first highly affine galactose trimer was described by Biessen et al. who used a TRIS-core to attach three galactose units on a PEG-linker at a distinct distance of 20 Å (Biessen et al. 1995). This concept was later re-used by Khorev et al. who synthesized trimeric galactose and *N*-acetylgalactose conjugates based on the same scaffold. In the latter case PEG_4_-linkers were used to keep the galactose residues at a distance of 18 Å. Counting atoms from the branching point of TRIS to the sugar implies that 14–16 atoms roughly correspond to a distance of 18–20 Å, which is reported to be the ideal distance in order to obtain optimal binding geometry to the receptor (Lee & Lee 2000). Our design considered this and resulted in NODAGA-TriGalactan. Comparison of the linker length with the above mentioned trimers can be found in Fig. 8.

In case of NODAGA-TriGalactan 15 atoms between the branching point and the galactose are implemented, corresponding to a distance of approximately 19 Å. Thus, we can assume that our design strategy is within the sweet spot of the needed spatial geometry. Indeed, this hypothesis is supported by the fact that the IC_50_ value we have found for ^nat^Ga-NODAGA-TriGalactan lays in the upper nanomolar range. However, the data also suggest that GSA is still more affine for the receptor than the trimer. This could be due to the fact that one molecule of ^99m^Tc-GSA presents about 35 galactose units. Thus, the probability that one of these sugar moieties takes part in an interaction with the ASGR is much higher than, if ultimately only three galactose units per molecule are present. Nevertheless, the IC_50_ data showed here possess a high variance and might only give more of a relation between two compounds than claim to be absolute values. One major problem with the affinity assay is, that it is based on freshly isolated mouse hepatocytes and for every assay, a new batch of cells has to be isolated. Therefore it cannot be excluded that environmental factors such as age or workup time might have influence on the amount of active receptors expressed and thus on the experimental outcome. However, as ASGR expression is by far the highest on primary hepatocytes (500,000 copies per cell vs. HepG2 76,000 copies per cell) (D’Souza & Devarajan 2015), we assumed that this assay design still gives the most accurate estimation of affinity values.

As a more sustainable alternative to freshly isolated hepatocytes, in vitro studies on mouse hepatic liver organoids were performed as well. These cellular systems have the advantage of providing functional organ-like structures in a dish without the need for laborious tissue isolation procedures. In contrast to conventional cell culture techniques, where one specific cell line is aligned in a 2D plane, hepatic organoids comprise of liver progenitor cells that can further differentiate in various functional cell types such as hepatocytes, Kupffer-cells or cholangiocytes in a 3D manner (Broutier et al. 2016). Therefore, liver organoids possess the ability to provide a more realistic in vitro model for organ function. Their utility in e.g. studying liver diseases has been reviewed recently (Harrison et al. 2021). Indeed, mouse hepatic organoids showed statistically significant interaction with ^68^Ga[Ga]-NODAGA-TriGalactan and presence of the ASGR was furthermore confirmed in fluorescence microscopy studies. Additionally, signals of BP-Fluor-647-TriGalactan were located inside the organoid sphere indicating internalization of the compound. However, as internalization of the ligand-receptor complex removes possible binding sites for the Anti-ASGR1 antibody, no co-localization of the receptor and the trimer was possible.

Less room for interpretation leave the biodistribution data, wherein ^99m^Tc-GSA shows almost double the liver uptake than the trimeric conjugate. Despite the overall lower liver uptake of [^68^Ga]Ga-NODAGA-TriGalactan, liver-to-organ ratios are higher for the trimer 30 and 60 min p.i. indicating a quick washout from non-target tissue and fast clearance from the blood pool. These findings are also in line with the imaging data, wherein TACs for liver and heart are well separated. Interestingly, this is contrary to ^99m^Tc-GSA, where kidney uptake as well as blood pool activity stayed at an almost constant level over the course of the experiment. One possible explanation could be the significantly higher molecular weight of GSA, which lays in the range of 80 kDa (Haubner et al. 2017). As renal cut-off is at about 30 kDa, ^99m^Tc-GSA exceeds by far the filtration limit and hence cannot be easily eliminated from the circulation (Kompella 1999). Furthermore, its intestinal elimination is much more pronounced, indicating a higher metabolic degradation and biliary elimination of ^99m^Tc-GSA after having been internalized by the hepatocyte. This phenomenon is significantly less evident for [^68^Ga]Ga-NODAGA-TriGalactan, which could be due to the much higher hydrophilicity of the trimer compared to the full size protein as well as the lower stability found for ^99m^Tc-GSA. Additionally, this finding is also in line with the low protein binding of the trimer, resulting in a quick clearance of any unbound ligand from the blood flow.

Despite the good pharmacokinetic properties of our first generation compound [^68^Ga]Ga-NODAGA-TriGalactan, further optimization might be beneficial in order to develop the most potent low molecular weight analogue for ^99m^Tc-GSA. One good example on how this can be achieved, has been shown quite recently by a group from Taiwan. Their molecule termed “HexaLac” is a poly-lysine based hexamer of lactose units, that can be labelled with either indium-111 for SPECT (Lee et al. 2011) or gallium-68 and fluorine-18 for PET (Yu et al. 2018, 2020). Instead of using the TRIS-based backbone they introduced a dendritic structure und hence, increased the interaction possibilities of the ligand with its receptor. Furthermore they exchanged galactose by lactose, which is a disaccharide consisting of galactose and glucose. As a result they found high affinity (IC_50_ = 9 nM) on rat hepatocytes and superior liver uptake (∼ 75% ID/g 120 min p.i.) in healthy BALB/c mice. In case of NODAGA-TriGalactan it would be possible to exchange galactose by *N*-acetylgalactose as the latter is known to have much higher affinity for the ASGR. By replacing NODAGA with NOTA other PET nuclides such as copper-64 (Pippin et al. 1991, Hoffman & Smith 2009) or fluorine-18 using the “AlF”-approach (Da Pieve et al. 2020) could be included into the structure and make this molecule also attractive for a centralized clinical routine supply.

## Conclusions

So far functional liver imaging with SPECT using ^99m^Tc-GSA has found its way into clinical routine only in Japan. Due to the unavailability of suitable tracers, clinical translation of this diagnostic tool hasn’t been realized in other countries, yet. In this article, we describe the successful development of a trimeric galactose conjugate that can easily be labelled with ^68^Ga-gallium and possesses good affinity for the ASGR. This receptor is specifically expressed on functional hepatocytes and hence, allows imaging of the functional liver reserve. [^68^Ga]Ga-NODAGA-TriGalactan showed favourable properties such as high hydrophilicity, good liver accumulation and fast washout from non-target organs resulting in excellent liver-to-background ratios. Structural optimization for example by exchanging galactose by *N*-acetylgalactose could further enhance liver uptake. Thus, [^68^Ga]Ga-NODAGA-TriGalactan marks a valuable step towards the development of low molecular weight tracers for the non-invasive determination of the ASGR status.

### Electronic supplementary material

Below is the link to the electronic supplementary material.

Supplementary Material 1

## Data Availability

The datasets generated during and/or analysed during the current study and not found in the supplementary information are available from the corresponding author on reasonable request.

## Abbreviations

AlF: Aluminium fluoride species
ASGR: Asialoglycoprotein receptor
ASOR: Asialoorosomucoid
CRD: Carbohydrate recognition domain
DIPEA: Diisopropylethylamine
DMF: Dimethylformamide
FDG: Fluorodesoxyglucose
Gal: Galactose
GalNAc: *N*-acetylgalactosamine
GSA: Galactosyl serum albumin
HATU: [O-(7-Azabenzotriazol-1-y)-N, NN’N’-tetramethyluronium-hexafluorophosphat
HOAt: 1-hydroxy-7-azabenzotriazol
HPLC: High performance liquid chromatography
IC_50_: Inhibitory constant
ID/g: Injected dose per gram
KOH: Potassium hydroxide
MeCN: Acetonitrile
MeOH: Methanol
MR: Magnetic resonance
NaOH: Sodium hydroxide
nat: Natural
NEt_3_: Triethylamine
NHS: *N*-hydroxy succinimide
NMR: Nuclear magnetic resonance
NOTA: 1,4,7-triazacyclononane-1,4,7-triacetic acid
NTG: NODAGA-TriGalactan
o.n.: Over night
p.i.: Post injection
PBS: Phosphate-buffered saline
PET: Positron emission tomography
SPECT: Single photon emission tomography
TAC: Time activity curve
*t*BuOH: *tert*Butanol
TFA: Trifluoroacetic acid
TIPS: Triisopropylsilan
TLC: Thin-layer chromatography
t_R_: Retention time
TRIS: Tris(hydroxymethyl)aminomethan
