# Supplementary material for: [68Ga]Ga-NODAGA-TriGalactan, a low molecular weight tracer for the non-invasive imaging of the functional liver reserve
Source: EJNMMI Radiopharm Chem. 2024 May 15;9:41. doi: 10.1186/s41181-024-00271-1 (PMC11096148; doi:10.1186/s41181-024-00271-1)
Supplement: Supplementary file 1 — Supplementary Material 1 [file 41181_2024_271_MOESM1_ESM.docx]

**Supporting Information**

[^68^Ga]Ga-NODAGA-TriGalactan, a low molecular weight tracer for the non-invasive imaging of the functional liver reserve

Maximilian A. Zierke^1^, Christine Rangger^1^, Kimia Samadikhah^2^, Marlene Panzer^3^, Stefanie Dichtl^4^, Nikolas Hörmann^5^, Doris Wilflingseder^4^, Andreas M. Schmid^2^, Roland Haubner^1,§^

^1^Department of Nuclear Medicine, Medical University Innsbruck, Anichstr. 35, 6020 Innsbruck, Austria

^2^Werner Siemens Imaging Center, Department of Preclinical Imaging and Radiopharmacy, Eberhard Karls University Tübingen, Röntgenweg 13, 73076 Tübingen, Germany

^3^Department of Internal Medicine I, Medical University Innsbruck, Anichstr. 35, 6020 Innsbruck, Austria

^4^Institute of Hygiene and Medical Microbiology, Medical University Innsbruck, Schöpfstr. 41, 6020 Innsbruck, Austria

^5^Department of Pharmaceutical Chemistry, Institute of Pharmacy, University of Innsbruck, Innrain 80-82, 6020 Innsbruck, Austria

^§^to whom correspondence should be addressed

[roland.haubner@tirol-kliniken.at](mailto:roland.haubner@tirol-kliniken.at)

**Table of content**

[**General Information** 3](#_heading=h.gjdgxs)

[**Synthesis of NODAGA-TriGalactan & BP-Fluor-647-TriGalactan** 4](#_heading=h.30j0zll)

[Boc-Tris(Propargyl)_3_ (**2**) 4](#_heading=h.1fob9te)

[Tris{PEG_3_-Gal(Ac_4_)}_3_ (**3**) 5](#_heading=h.3znysh7)

[GABA-Tris{PEG_3_-Gal(Ac_4_)}_3_ (**4**) 6](#_heading=h.2et92p0)

[NODAGA-TriGalactan (**5**) 7](#_heading=h.tyjcwt)

[BP-Fluor-647-TriGalactan (**6**) 10](#_heading=h.3dy6vkm)

[**Preparation of [^99m^Tc]Tc-GSA** 12](#_heading=h.1t3h5sf)

[**Preparation of [^125^I]I-ASOR** 12](#_heading=h.4d34og8)

[***In vitro* binding studies on murine hepatocytes** 14](#_heading=h.2s8eyo1)

[**Mouse hepatic organoid culture** 15](#_heading=h.17dp8vu)

[**Additional Tables** 16](#_heading=h.3rdcrjn)

[**References** 17](#_heading=h.26in1rg)

# **General Information**

All reagents were obtained from VWR International GmbH (Vienna, Austria) or Merck (Darmstadt, Germany) and used as supplied without further purification. Human α_1_-Glycoprotein was obtained from Merck. NODAGA-NHS ester (2,2′-(7-(1-carboxy-4-((2,5-dioxopyrrolidin-1-yl)oxy)-4-oxobutyl)-1,4,7-triazonane-1,4-diyl)diacetic acid) was purchased from Chematech (Dijon, France). 2-{2-[2-azidoethoxy]ethoxy}ethyl β-d-galactopyranoside and propargyl bromide were obtained from Santa Cruz Biotech (Dallas, Texas, USA). DTPA-conjugated galactosyl human serum albumin (GSA) was a kind gift from Nihon Medi-Physics (Tokyo, Japan) and was supplied as a freeze-dried technetium kit formulation. The kit was dissolved with water and aliquoted in 130 µg portions, which were then used for ^99m^Tc-labelling.

The ^99^Mo/^99m^Tc generator was purchased from THP Medical Products (Vienna, Austria) with a nominal activity of 8.6 GBq and was eluted with 10 ml isotonic NaCl solution. The ^68^Ge/^68^Ga generator was purchased from Eckert & Ziegler (IGG 100, Berlin, Germany) with a nominal activity of 1.85 GBq and was eluted with 0.1 N HCl (GMP grade, Rotem Industries Ltd., Mishem Yamin D. N. Arava, Israel). ^125^I-iodine was supplied by Perkin Elmer (Waltham, Massachussetts, USA) at an activity of 37 MBq.

Radio thin-layer chromatography (TLC) was performed with iTLC-SG stripes (Agilent Technologies, Santa Clara, California, USA) using either 0.1 M Na-Citrate or 1 M Na-Acetate/MeOH (1:1, v/v) as eluents. Read out of the stripes was done using a ScanRAM radio-TLC scanner (LabLogic, Broomhill, Sheffield, UK). Radioactivity of the samples was quantified using a 2480 Wizard^2^ 3” γ-counter (Perkin Elmer (Waltham, Massachussetts, USA).

Analytical reversed-phase (RP) HPLC was performed on an UltiMate 3000 HPLC system (Thermo Fisher, Waltham, Massachussetts, USA) equipped with a quarternary RS Pump, Autosampler, RS Column Compartment, Variable Wavelength Detector and a GabiStar (Raytest, Straubenhardt, Germany) radio detector. All devices were controlled with the Chromeleon 7 software. Degassed water (solvent A) and acetonitrile (solvent B) both containing 0.1% (v/v) of trifluoroacetic acid (TFA) were used as eluents at flow rates of 1 mL/min. The columns in use were either a ReproSil Pur C_18_ AQ 150 × 4.6 mm, 5 µm, 120 Å (Dr. Maisch, Ammerbuch-Entringen, Germany), a Jupiter C_18_ 150 × 4.6 mm, 5 µm, 300 Å (Phenomenex, Richmond, Canada) or a Nucleosil 120-3 C_18_ 150 × 4.6 mm (SRD, Wien, Austria).

Semipreparative RP-HPLC was performed on a GILSON (Middleton, WI, USA) 322 chromatography system equipped with a GILSON UV/VIS-155D multi-wavelength UV detector and fraction collector system. Degassed water and acetonitrile both containing 0.1% (v/v) of TFA were used as solvents at flow rates of 10 mL/min. The column in use was a ReproSil Pur C_18_ AQ 250 x 20 mm, 5 µm, 120 Å.

Mass spectra were recorded on a Microflex Benchtop MALDI-TOF MS (Bruker, Billerica, Massachussetts, USA) in positive or negative ionization mode. Samples were mixed 1:1 (v/v) with a saturated solution of α-cyano-4-hydroxycinnamic acid containing 0.1% TFA placed on a steel target matrix and dried under a stream of argon. Additional mass spectra were recorded on a LTQ Velos ESI-MS device (Thermo Fisher Scientific).

NMR spectra were recorded on an Avance 4 Neo spectrometer (Bruker) at 700 MHz (^1^H) and 176 MHz (^13^C). Chemical shifts are given in parts per million (ppm) and calibrated on the residual solvent signal. As deuterated NMR solvents were either used chloroform-d^1^ or methanol-d^4^ (Eurisotop®, Saint-Aubin, France). Coupling constants (*J*) are reported in Hertz (Hz) and multiplicities are denoted as follows: s – singlet, d – doublet, t – triplet, q – quartet, m – multiplet.

# **Synthesis of NODAGA-TriGalactan and BP-Fluor-647-TriGalactan**

## Boc-TRIS(Propargyl)_3_ (**2**)

To a 100 mL round bottom flask 500 mg (2.3 mmol, 1 eq) of Boc-protected Tris(hydroxymethyl)aminomethan (TRIS) (**1**) was added as a solid and dissolved in 6 mL of dry N,N-Dimethylformamide (DMF). The solution was cooled on ice followed by addition of 1.5 mL of propargylbromid in toluene (80 %, 13.9 mmol, 6.2 eq) as well as 1 g (17.9 mmol, 7.9 eq) of fine powdered KOH. The reaction mixture was stirred on ice for 15 min and was allowed to warm up to room temperature afterwards. For workup, 20 mL of ethyl acetate were added and the resulting solution was transferred into a separation funnel where the organic layer was washed three times with 20 mL of distilled water. The organic layer was separated and the aqueous extracts were washed twice with 20 mL of ethyl acetate. The combined organic fractions were dried over NaSO_4_, filtered and all volatiles removed *in vacuo* yielding the raw product as a yellowish oil, which was subsequently purified using column chromatography with a gradient of *n*-hexane/ethyl acetate (90/10 – 40/60) (v/v). The product containing fractions were pooled and all volatiles were removed *in vacuo* to yield the product as a yellowish oil that solidified upon storage in the refrigerator.

**^1^H-NMR** (CDCl_3_): δ (ppm) = 4.91 (s, 1 H, NH), 4.13 (m, 6H, CH_2_-C≡C), 3.75 (s, 6 H, O-CH_2_), 2.48 (t, 3 H, ^4^*J* = 1.8 Hz, C≡CH), 1.41 (s, 9H, CH_3_).

**MALDI-MS** (m/z) **=** 336.3 [M+H]^+^, 358.3 [M+Na]^+^.

## TRIS{PEG_3_-Gal(Ac_4_)}_3_ (**3**)

Compound **2** (24.9 mg, 74.2 µmol, 1 eq) was weighed into a 15 mL Falcon tube and a solution of 135 mg (267 µmol, 3.6 eq) 2-{2-[2-azidoethoxy]ethoxy}ethyl β-d-galactopyranoside in 600 µL MeOH was added. The reaction was started by subsequent addition of an aqueous solution of Cu(OAc)_2_*H_2_O (17.8 mg, 89.1 µmol, 1.2 eq) and sodium ascorbate (586 mg, 3 mmol, 40 eq) and the resulting mixture was heated on a water bath at 60°C for 1 h. For workup, all volatiles were removed *in vacuo* and for Boc-deprotection the residue was resuspended in 4 mL of TFA/TIPS/H_2_O (95/2.5/2.5) (v/v/v) and incubated for 1 h at room temperature. After removal of TFA under a stream of argon, the raw product was dissolved in 1.6 mL of H_2_O/30% MeCN (v/v) and substracted to semipreparative RP-HPLC (30-50 % B in 20 min). The product was obtained as a colourless oil (27 mg 14.5 µmol, 20 %).

**Analytical HPLC** (ReproSil Pur, 20-80 % B in 15 min, 1.0 mL/min) t_R_ = 13.6 min (70 % B)

**MALDI-MS** (m/z) = 1750.2 [M]^+^, 1772.2 [M+Na]^+^.

## GABA-TRIS{PEG_3_-Gal(Ac_4_)}_3_ (**4**)

In a V-shaped vial HOAt (3.95 mg, 29.0 µmol, 2 eq), HATU (11.0 mg, 29.0 µmol, 2 eq), and Boc-GABA (5.89 mg, 29.0 µmol, 2 eq) were dissolved in 100 µL of anhydrous DMF and DIPEA (15.2 µL, 87.0 µmol, 6 eq). After 10 minutes of pre-activation this solution was added dropwise to a solution of compound **3** (27.0 mg, 14.5 µmol, 1 eq) in 350 µL dry DMF and 15.2 µL (87.0 µmol, 6 eq) DIPEA. The reaction mixture was left at room temperature for 2 h before 500 µL water were added to deactivate the remaining coupling agents. The solution was transferred to a 10 mL round bottom flask and all volatiles were removed *in vacuo*. For Boc-deprotection the residue was resuspended in 1 mL TFA/TIPS/H_2_O (95/2.5/2.5) (v/v/v) and incubated for 1 h at room temperature. After removal of TFA under a stream of argon the raw product was dissolved in 660 µL H_2_O/30 % MeCN and substracted to semipreparative RP-HPLC (30-45 % B in 20 min). The product was obtained as colourless oil (15.9 mg, 8.16 µmol, 56%).

**Analytical HPLC** (ReproSil Pur, 20-80 % B in 15 min, 1.0 mL/min) t_R_ = 11.2 min (61 % B).

**MALDI-MS** (m/z) = 1835.8 [M]^+^, 1857.2 [M+Na]^+^.

## NODAGA-TriGalactan (**5**)

*(R)-*NODAGA-NHS (7.17 mg, 9.79 µmol, 1.2 eq) was dissolved in 100 µL anhydrous DMF and DIPEA (4.27 µL, 24.5 µmol, 3 eq). This mixture was then added dropwise to a solution of compound **4** (15.9 mg, 8.16 µmol, 1 eq) in 300 µL anhydrous DMF and DIPEA (8.53 µL, 49.0 µmol, 6 eq). The pH was adjusted to 9 by addition of another 3 eq DIPEA and the mixture was stirred over night at room temperature. For subsequent de-acetylation the crude reaction mixture was reduced to dryness and the residue was dissolved in 3 mL NEt_3_/MeOH/H_2_O (1:6:2) (v/v/v). After 12 hours all volatiles were removed *in vacuo* and the raw product was purified via semi-preparative RP-HPLC (12 % B isocratic). Lyophilisation yielded 4.48 mg (2.34 µmol, 29 %) of a colourless solid.

**Analytical HPLC** (ReproSil Pur, 5-60 % B in 15 min, 1.0 mL/min) t_R_ = 7.2 min (28 % B).

**MALDI-MS** (m/z) = 1689.7 [M+H]^+^.

**^1^H-NMR** (700 MHz, CD_3_OD): δ (ppm) = 8.02 (s, 3 H, k), 4.59 (t, 6 H, ^3^*J* = 5.1 Hz, l), 4.57 (s, 6 H, j), 4.26 (d, 3 H, ^3^*J* = 7.6 Hz, o), 3.98 (ddd, 3 H, ^3^*J* = 11.0 Hz, 5.3 Hz, 3.6 Hz, n), 3.90 (t, 6 H, *^3^J* = 5.1 Hz, m), 3.84 (dd, 3 H, ^3^*J* = 3.4 Hz, 1.1 Hz, r), 3.79 (s, 1 H, c), 3.77 (s, 6 H, i), 3.74 (dd, 7 H, *^3^J*= 14.0 Hz, 6.1 Hz, a/q), 3.72-3.68 (m, 5 H, n), 3.63 (dt, 6 H, *^3^J* = 5.1 Hz, 3.6 Hz, n), 3.61 (s, 10 H, n), 3.55-3.51 (m, 9 H, p/t), 3.48 (dd, 3 H, *^3^J* = 9.7 Hz, 3.4 Hz, s), 3.19-2.90 (m, 12 H, b), 2.47-2.38 (m, 2 H, e), 2.22 (t, 2 H, *^3^J* = 7.5 Hz, h), 2.16-2.11 (m, 1 H, d), 1.98-1.96 (m, 1 H, d), 1.73 (p, 2 H, ^3^*J* = 7.2 Hz, g), 1.37 (t, 2 H, *^3^J* = 6.2 Hz, f).

**^13^C-NMR** (176 MHz, CD_3_OD): δ (ppm) = 175.8 (C-v/w), 175.3 (C-y/z), 145.7 (C-u), 126.0 (C-k), 105.1 (C-o), 76.7 (C-q), 74.9 (C-s), 72.5 (C-p), 71.5 (C-n), 71.4 (C-n), 70.4 (C-m), 70.3 (C-r), 69.7 (C-i), 69.4 (C-c), 65.3 (C-x), 62.6 (C-a/n/t), 61.4 (C-j), 51.2 (C-b), 35.0 (C-h), 33.7 (C-e), 31.7 (C-f), 27.0 (C-d), 25.0 (C-g).


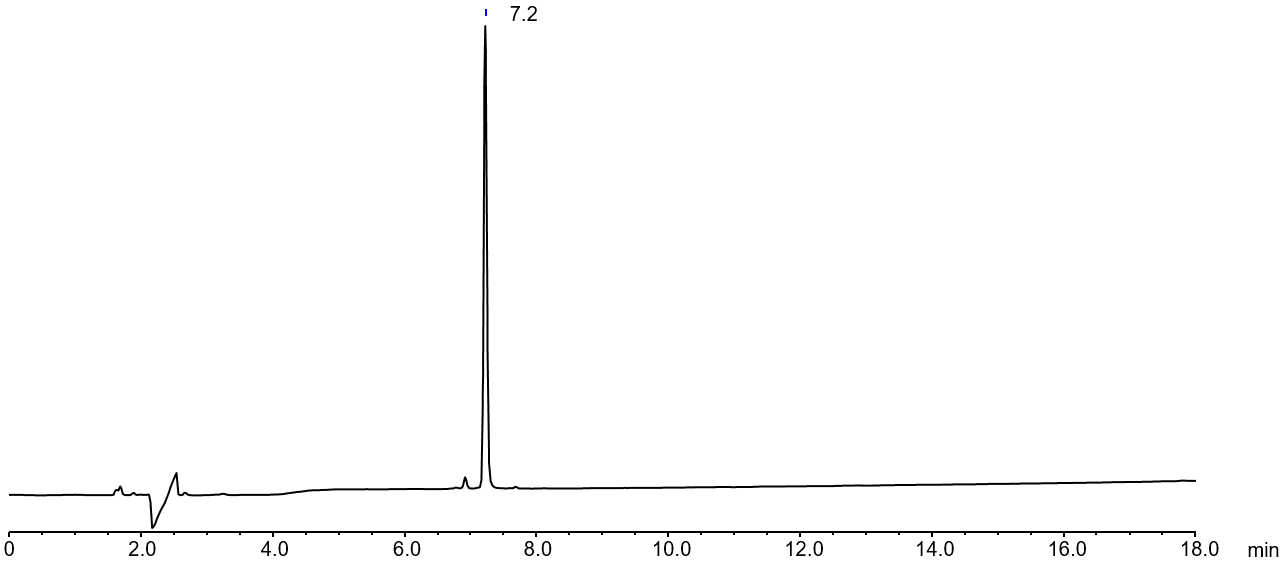


RP-HPLC chromatogram of NODAGA-TriGalactan at λ = 220 nm.


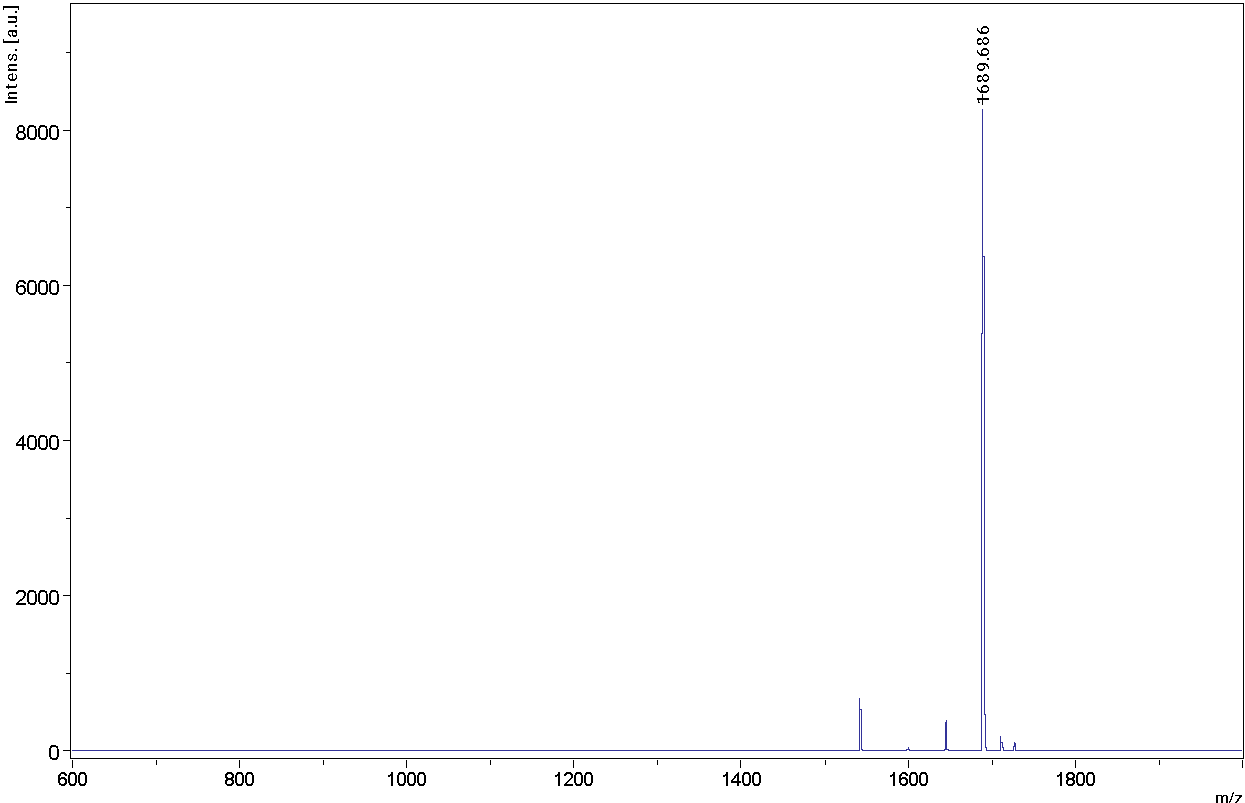


Mass spectrum of NODAGA-TriGalactan.

^1^H-NMR spectrum of NODAGA-TriGalactan.

^13^C{^1^H}-NMR spectrum of NODAGA-TriGalactan.

HSQC-NMR spectrum of NODAGA-TriGalactan showing cross peaks between 0.5-9.0 ppm (^1^H) and 25-125 ppm ^13^C.

{^1^H, ^1^H}-COSY spectrum of NODAGA-TriGalactan showing correlations between 1.0-5.0 ppm.

## BP-Fluor-647-TriGalactan (**6**)

Compound **4** (4.77 mg, 2.45 µmol, 1.0 eq) was treated over night with 1 mL of a cocktail of NEt_3_/MeOH/H_2_O (1:6:2) (v/v/v). All volatiles were removed *in vacuo* and the deprotected trimer was purified via semi-preparative HPLC (12-17 % B in 25 min). This intermediate was lyophilised and reconstituted in 50 µL of dry DMF. To this solution BP-Fluor-647 NHS-Ester (1 mg, 978 nmol, 1.1 eq) in 150 µL of dry DMF was added. The pH was adjusted to 9 by addition of DIPEA (1.4 µL) and the reaction was allowed to run for 2 hours at room temperature. The reaction mixture was diluted with 800 µL Millipore water and substracted to semi-preparative HPLC (12-17 % B in 25 min). After lyophilisation BP-Fluor-647-TriGalactan was obtained as a blue solid (320 µg, 133 nmol, 15 %).

**Analytical HPLC** (ReproSil Pur, 5-60 % B in 15 min, 1.0 mL/min) t_R_ = 7.3 min (28 % B).

**MALDI-MS** (m/z) = 2171.5 [M-H]^-^, 2234.4 [M+Na+K]^-^, 2297.5 [M+2Na+2K]^-^, 2360.1 [M+3Na+3K]^-^.


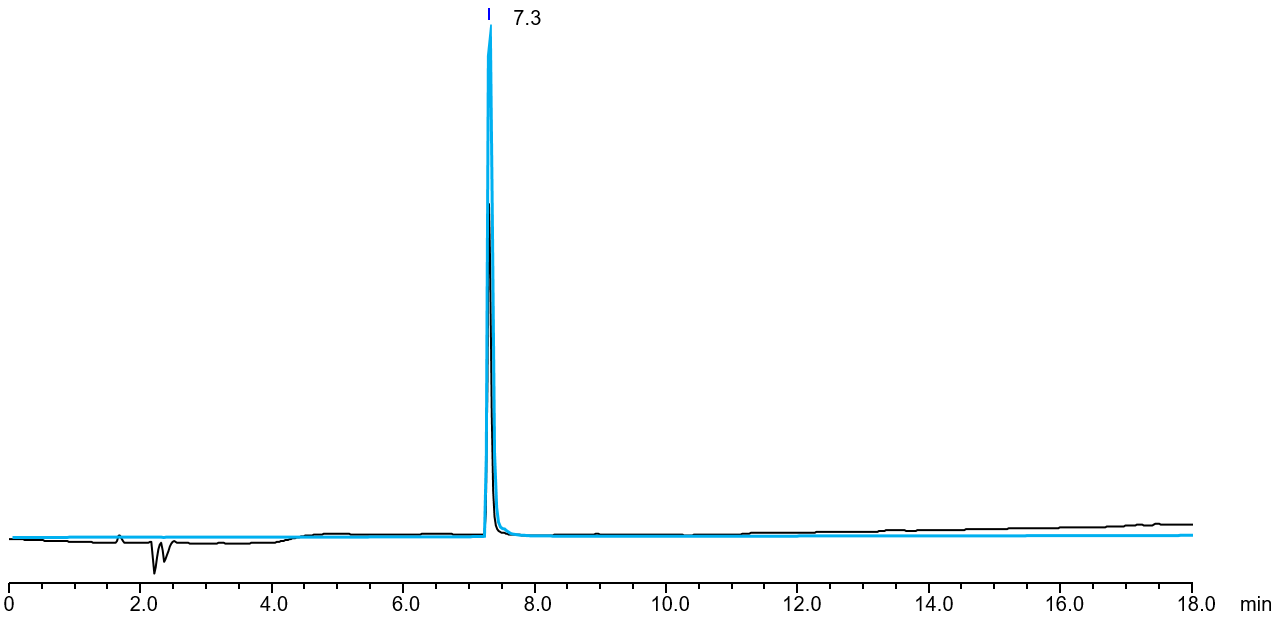


RP-HPLC chromatogram of BP-Fluor-647-TriGalactan at λ = 220 nm (black) and 650 nm (cyan).


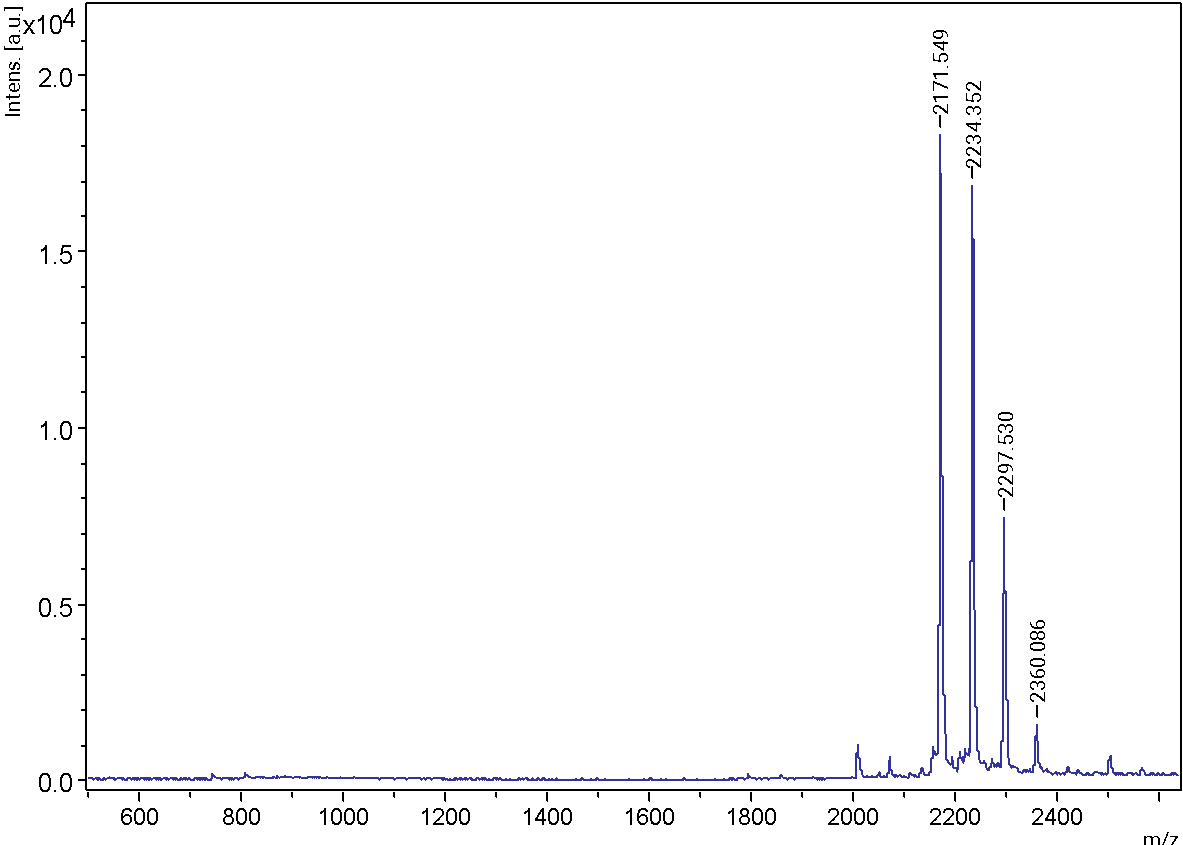


Mass spectrum of BP-Fluor-647-TriGalactan.

# **Preparation of [^99m^Tc]Tc-GSA**

Preparation of the clinically established reference ligand [^99m^Tc]Tc-GSA was done according to reference [1]. In brief, 130 µg of GSA were dissolved in 100 µL Millipore water and 100-300 µL ^99m^Tc-Pertechnetate solution (approx. 200 MBq) were added as well as 4 µL (22 mM, 88 nmol) freshly prepared SnCl_2_ solution in 0.1 M HCl. The mixture was gently shaken (300 1/min) and incubated for 10 min at 37°C. Completion of the labelling reaction was monitored with radio-HPLC (Jupiter, Gradient: 10-60 % B in 15 min). The labelled compound was then diluted with PBS without further purification. For *in vivo* studies the ^99m^Tc-pertechnetate solution was prediluted with 0.9 % saline to approx. 15 MBq/100 µL in order to reach the same molar activity as for the ^68^Ga-labelled tracer.

**Radio-HPLC** (Jupiter, 10-60 % B in 15 min, 1.0 mL/min) t_R_ = 9.0 min (36 % B).


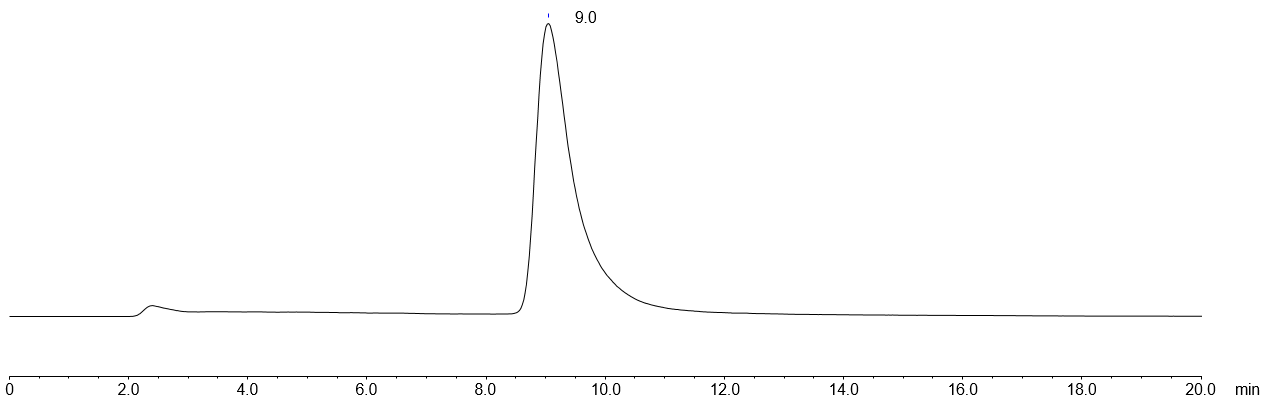


Radio-HPLC of ^99m^Tc-GSA.

# **Preparation of [^125^I]I-Asialoorosomucoid (ASOR)**

Preparation of the standard ligand for *in vitro* studies was done according to a previously published procedure [2]. Briefly, human ɑ_1_-Glycoprotein (10 mg) was desialylated in 700 µL 0.1 M H_2_SO_4_ by heating to 80 °C for 1 hour. The solution was neutralized with 0.1 M NaOH and was passed through an Amicon^®^ (Merck, Darmstadt, Germany) Ultra 0.5 centrifuge filter (MWCO 3 kDa). The filter unit was spun at 3000 rpm for 1 hour. The filter cake was reconstituted with 500 µL of water and the filter was spun again at 3000 rpm for 30 min. This procedure was repeated three times before the supernatant was transferred to a 1.5 mL Protein Low-Bind tube and lyophilized yielding 6.7 mg of a white solid. Removal of the sialic acid groups from the protein was confirmed by MS analysis as described in literature and showed high abundance of double (>96%) and triple (97%) deprotected peptide fragments [3]. The desialylated protein was aliquoted in 500 µg portions and stored at -20 °C until usage.

For iodination 100 µg ASOR were transferred to an Iodo-Gen^®^ (150 µg, Merck) coated Eppendorf tube containing 100 µL of PBS followed by addition of approx. 5 MBq ^125^I-iodine. The vial was gently vortexed and the reaction proceeded over the course of 20 min at room temperature. For workup the labelling solution was loaded onto a preconditioned PD-10 (GE Healthcare, Chicago, Illinois, USA) desalting column (bed size 5 × 1.2 cm) containing Sephadex-G25 M. The labelled protein was eluted in 500 µL fractions using PBS and the activity content of each fraction was quantified in a γ-counter. The fractions containing the highest activities were combined (vials 6-7) yielding 3.8 MBq (RCP ≥ 99%) of the labelled compound.

**Radio-HPLC** (Jupiter, 10-60 % B in 15 min, 1.5 mL/min) t_R_ = 13.5 min (51 % B).


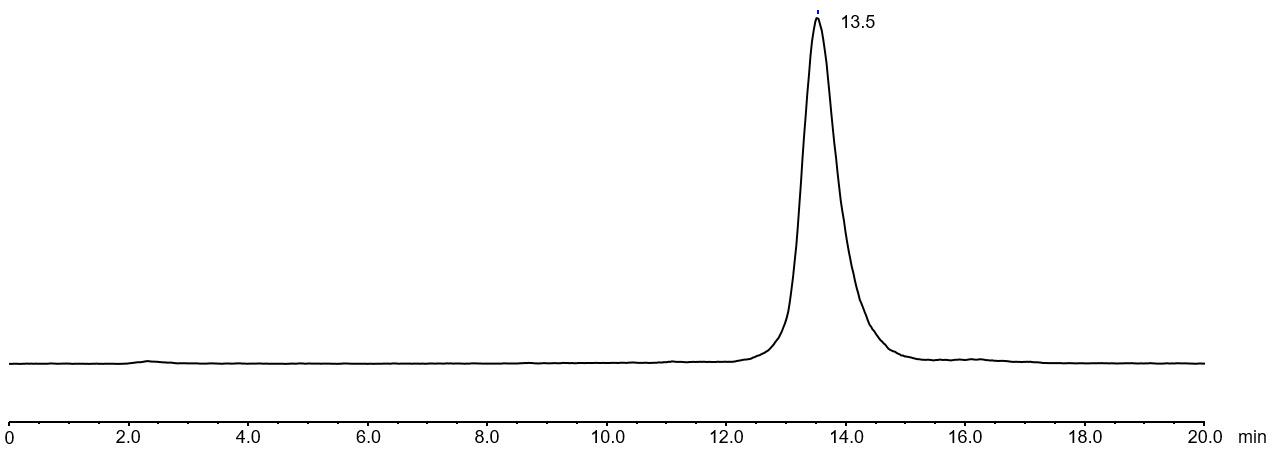


Radio-HPLC of [^125^I]I-ASOR.

# ***In vitro* binding studies on murine hepatocytes**

Isolation of murine hepatocytes followed the collagenase perfusion method published by Seglen [4]. Therefore, C57BL/6 mice were euthanized by cervical dislocation and subsequently the peritoneum was opened. A catheter (BD Neoflon^TM^, Heidelberg, Germany) was inserted into the vena cava inferior and fixated in place. Aorta and vena cava were disconnected with a micro surgical clamp and the vena portae hepatis was cut open. The catheter was connected to a motor pump and the liver tissue was perfused with 70 mL perfusion buffer (140 mM NaCl, 10 mM KCl, 10 mM HEPES, 5.5 mM NaOH, 100 µM EGTA, pH 7.4; 12 mL/min), followed by 20 mL collagenase buffer (66 mM NaCl, 12 mM KCl, 5 mM CaCl_2_, 100 mM HEPES, 50 mM NaOH, pH 7.4; 12 mL/min) and 40 mL liberase buffer (collagenase buffer + 750 µg Liberase^TM^, Roche, Basel, Switzerland; 8 mL/min). Liver was taken out and directly transferred to a petri dish (ø = 10 cm) filled with 10 mL of ice-cold L-15 medium (Merck). Next steps were performed in a biological safety cabinet. The gallbladder was removed and liver cells were carefully extracted from the organ capsule using a cell scraper. The resulting cell suspension was filtered through a 100 µm cell strainer (Corning^®^, Corning, NY, USA) into a 50 mL Falcon tube and the dish was washed with 10 mL of hepatocyte wash medium. Cells were centrifuged (3 min, 29 rcf, 4°C, slow brake), the supernatant was removed and the pellet was washed with 30 mL of hepatocyte wash medium (Gibco, Thermofisher Scientific). Next, cells were resuspended in 30 mL of percoll solution (Merck) and substracted to an isodense centrifugation step (10 min, 45 rcf, 4°C, slow brake). The supernatant containing dead and non-parenchymal cells was carefully removed and the pellet was thoroughly washed with hepatocyte wash medium (3 × 30 mL). Hepatocytes were resuspended in 20 – 30 mL of William’s E + Glutamaxx + 10 % FCS + 1 % Penicillin/Streptomycin (Gibco, Thermofisher Scientific) and counted, typically yielding 30 – 40 × 10^6^ cells/animal.

For determination of inhibitory constant (IC_50_) values, freshly isolated hepatocytes were seeded overnight into a 24-well collagen-I coated plate (Corning^®^) at a density of 70,000 cells/well. Cells were washed once with 300 µL of PBS and 200 µL of Williams E + 5 mM CaCl_2_ + 2 % BSA was added. The cells were put on ice for 10 min before 25 µL of a dilution series of either ^nat^Ga-NODAGA-TriGalactan or GSA in PBS (10^-4^ – 10^-10^ M) were added. With addition of 25 µL radioligand ([^125^I]I-Asialoorosomucoid; 5 nM; 2.4 kBq per well), ligand dilutions were ranging from 10^-5^ – 10^-11^ M. Cells were incubated for 1 hour on ice before the supernatant was removed. Cells were washed with 300 µL of ice cold Williams E + 5 mM CaCl_2_ + 2 % BSA. Supernatant and wash fractions were combined. Cell bound activity was released by addition of 200 µL 1 M NaOH for 10 min. Lysates were removed and the wells washed again with 200 µL of 1 M NaOH. The activity content in the lysates was quantified in a γ-counter and the values plotted in excel. Fitting of the sigmoidal binding curve and the calculation of the IC_50_ value was done with excel’s solver plugin.

# **Mouse hepatic organoid culture**

Mouse hepatic organoids (Stemcell Technologies, Vancouver, Canada) were cultivated in HepatiCult^®^ organoid growth medium supplemented with 1 % Penicillin/Streptomycin + l-Glutamine (Thermofisher Scientific, Waltham, Massachussetts, USA) embedded in a Geltrex^TM^ (Thermofisher Scientific) LDEV-free reduced growth factor basement membrane matrix. Medium was replaced every 3-4 days and organoids were passaged every 7-10 days. For passaging, matrix domes were mechanically disrupted and the cell suspension was transferred into a 15 mL falcon tube. After centrifugation (300 rcf, 5 min, 4°C) the supernatant was removed and cells were treated with 500 µL of TrypLE^TM^ express enzyme for 8 min at 37°C. After addition of 500 µL fresh HepatiCult^®^ organoid growth medium, cells were spun down (300 rcf, 5 min, 4°C) and the supernatant was removed. Cells were resuspended in a mixture of organoid growth medium/geltrex (9/21) (v/v) on ice and domes were seeded out in a six-well plate. Upon polymerization of the matrix, domes were overlaid with 2 mL of organoid growth medium and the plate was retransferred to the incubator (37°C, 5% CO_2_, humidified atmosphere)

ASGR-binding studies require organoids from a matrix-free environment. Consequently, organoids were harvested as mentioned above and additionally, treated with 5 mM EDTA/PBS (pH 8) on ice for 90 min. Cells were spun down (300 rcf, 5 min, 4°C), the supernatant was removed and the pellet was washed once with sterile PBS. Organoids were resuspended in HepatiCult organoid growth medium and transferred to a 12-well low attachment plate without addition of geltrex. Medium was replaced every 2 days and organoids were kept in suspension culture for at least 7 days before usage.

# **Additional Tables**

**Table S1**. Biodistribution of [^68^Ga]Ga-NODAGA-TriGalactan (n=3) and ^99m^Tc-GSA (n=3) in healthy BALB/c mice. Data are expressed as a percentage of the injected dose per gram (% ID/g), mean value ± standard deviation.

| **[^68^Ga]Ga-NODAGA-TriGalactan** | | | | | | | **^99m^Tc-GSA** | | |  |
| --- | --- | --- | --- | --- | --- | --- | --- | --- | --- | --- |
|  | | | | | | **blocking** |  |  |  | |
|  | 10 min | | | 30 min | 60 min | 30 min | 10 min | 30 min | 60 min | |
| **Blood** | | 3.3 ± 0.2 | 0.89 ± 0.09 | | 0.34 ± 0.04 | 1.19 ± 0.33 | 3.13 ± 0.15 | 2.76 ± 0.57 | 1.98 ± 0.10 | |
| **Spleen** | | 0.78 ± 0.05 | 0.27 ± 0.02 | | 0.16 ± 0.02 | 0.32 ± 0.03 | 1.37 ± 0.16 | 1.42 ± 0.39 | 1.18 ± 0.20 | |
| **Pancreas** | | 0.87 ± 0.12 | 0.31 ± 0.05 | | 0.19 ± 0.09 | 0.45 ± 0.01 | 0.64 ± 0.02 | 0.48 ± 0.07 | 0.53 ± 0.17 | |
| **Stomach** | | 1.42 ± 0.23 | 1.05 ± 0.08 | | 0.92 ± 0.04 | 0.68 ± 0.11 | 0.91 ± 0.01 | 1.61 ± 0.20 | 2.43 ± 1.45 | |
| **Intestine** | | 1.13 ± 0.07 | 1.95 ± 0.17 | | 2.99 ± 0.43 | 0.77 ± 0.19 | 1.10 ± 0.05 | 6.41 ± 1.05 | 9.80 ± 1.61 | |
| **Kidneys** | | 8.15 ± 0.54 | 2.83 ± 0.18 | | 1.51 ± 0.10 | 4.19 ± 0.29 | 7.35 ± 0.51 | 10.5 ± 2.00 | 9.21 ± 0.21 | |
| **Liver** | | 33.4 ± 0.88 | 32.2 ± 3.70 | | 27.7 ± 3.12 | 14.6 ± 0.15 | 58.1 ± 6.68 | 51.3 ± 2.78 | 46.3 ± 5.88 | |
| **Heart** | | 1.18 ± 0.08 | 0.40 ± 0.03 | | 0.17 ± 0.00 | 0.44 ± 0.03 | 1.47 ± 0.07 | 1.08 ± 0.16 | 0.81 ± 0.07 | |
| **Lung** | | 2.14 ± 0.13 | 0.74 ± 0.03 | | 0.38 ± 0.10 | 0.97 ± 0.04 | 2.04 ± 0.11 | 1.64 ± 0.20 | 1.38 ± 0.11 | |
| **Muscle** | | 0.77 ± 0.02 | 0.23 ± 0.03 | | 0.17 ± 0.02 | 0.43 ± 0.20 | 0.60 ± 0.04 | 0.45 ± 0.06 | 0.39 ± 0.00 | |
| **Femur** | | 0.87 ± 0.52 | 0.22 ± 0.02 | | 0.26 ± 0.19 | 0.37 ± 0.16 | 1.54 ± 0.18 | 3.64 ± 1.98 | 2.48 ± 0.48 | |

**Table S2.** Liver-to-organ ratios of [^68^Ga]Ga-NODAGA-TriGalactan and ^99m^Tc-GSA in healthy BALB/c mice.

| **[^68^Ga]Ga-NODAGA-TriGalactan** | | | | | | **^99m^Tc-GSA** | | |  |
| --- | --- | --- | --- | --- | --- | --- | --- | --- | --- |
|  | | | | | |  |  |  | |
|  | **10 min** | | | **30 min** | **60 min** | **10 min** | **30 min** | **60 min** | |
| **Blood** | | 10.2 ± 0.8 | 35.4 ± 1.1 | | 85.5 ± 19 | 18.9 ± 1.2 | 17.5 ± 3.1 | 23.1 ± 1.5 | |
| **Spleen** | | 43.4 ± 6.8 | 121 ± 5.6 | | 177 ± 36 | 40.6 ± 2.7 | 32.8 ± 9.6 | 41.8 ± 3.5 | |
| **Pancreas** | | 38.7 ± 3.5 | 101 ± 10.8 | | 162 ± 44 | 91.8 ± 6.4 | 111 ± 20 | 99.6 ± 18 | |
| **Stomach** | | 24.1 ± 3.7 | 46.2 ± 23 | | 63.8 ± 40 | 59.8 ± 8.5 | 37.2 ± 11 | 30.3 ± 18 | |
| **Intestine** | | 29.7 ± 1.4 | 17.9 ± 4.5 | | 9.50 ± 2.3 | 57.0 ± 7.0 | 6.50 ± 0.6 | 4.60 ± 1.3 | |
| **Kidneys** | | 4.12 ± 0.4 | 10.7 ± 0.9 | | 18.3 ± 0.9 | 7.60 ± 0.5 | 4.70 ± 0.7 | 4.90 ± 0.6 | |
| **Heart** | | 28.8 ± 4.5 | 79.0 ± 4.6 | | 183 ± 58 | 40.4 ± 2.6 | 44.7 ± 7.3 | 54.1 ± 12 | |
| **Lung** | | 15.7 ± 1.4 | 40.5 ± 1.7 | | 75.0 ± 11 | 27.8 ± 4.2 | 30.2 ± 1.4 | 34.9 ± 2.2 | |
| **Muscle** | | 43.2 ± 1.3 | 135 ± 9.5 | | 173 ± 39 | 104 ± 9.6 | 114 ± 13 | 128 ± 18 | |
| **Femur** | | 49.5 ± 21 | 143 ± 12 | | 152 ± 64 | 31.8 ± 8.3 | 14.1 ± 3.5 | 19.8 ± 1.6 | |

# **References**

1. Kudo M, Washino K, Yamamichi Y, Ikekubo K. Synthesis and radiolabeling of galactosyl human serum albumin. Methods Enzymol 1994;247:383-394.

2. Li Y, Huang G, Diakur J, Wiebe LI. Targeted delivery of macromolecular drugs: asialoglycoprotein receptor (ASGPR) expression by selected hepatoma cell lines used in antiviral drug development. Curr Drug Deliv 2008;5:299-302.

3. Imre T, Schlosser G, Pocsfalvi G, Siciliano R, Molnár-Szöllősi É, Kremmer T, et al. Glycosylation site analysis of human alpha-1-acid glycoprotein (AGP) by capillary liquid chromatography—electrospray mass spectrometry. Journal of Mass Spectrometry 2005;40:1472-1483.

4. Seglen PO. Preparation of isolated rat liver cells. Methods Cell Biol 1976;13:29-83.
